# PC3T: a signature-driven predictor of chemical compounds for cellular transition

**DOI:** 10.1038/s42003-023-05225-y

**Published:** 2023-09-27

**Authors:** Lu Han, Bin Song, Peilin Zhang, Zhi Zhong, Yongxiang Zhang, Xiaochen Bo, Hongyang Wang, Yong Zhang, Xiuliang Cui, Wenxia Zhou

**Affiliations:** 1 Beijing Institute of Pharmacology and Toxicology, 100850 Beijing, China; 2 State Key Laboratory of Toxicology and Medical Countermeasures, Beijing, China; 3 Department of Pancreatic Surgery, Changhai Hospital, Second Military Medical University, 200438 Shanghai, China; 4 National Center for Liver Cancer, Eastern Hepatobiliary Surgery Hospital, Naval Medical University, 200438 Shanghai, China; 5 Fudan University Shanghai Cancer Center, Department of Oncology, Shanghai Medical College, Fudan University, 200032 Shanghai, China; 6 Department of Bioinformatics, Institute of Health Service and Transfusion Medicine, 100850 Beijing, China; 7 Institute for Regenerative Medicine, Shanghai East Hospital, Shanghai Key Laboratory of Signaling and Disease Research, Frontier Science Center for Stem Cell Research, School of Life Sciences and Technology, Tongji University, Shanghai, China

**Keywords:** Systems analysis, Predictive medicine, Reprogramming

## Abstract

Cellular transitions hold great promise in translational medicine research. However, therapeutic applications are limited by the low efficiency and safety concerns of using transcription factors. Small molecules provide a temporal and highly tunable approach to overcome these issues. Here, we present PC3T, a computational framework to enrich molecules that induce desired cellular transitions, and PC3T was able to consistently enrich small molecules that had been experimentally validated in both bulk and single-cell datasets. We then predicted small molecule reprogramming of fibroblasts into hepatic progenitor-like cells (HPLCs). The converted cells exhibited epithelial cell-like morphology and HPLC-like gene expression pattern. Hepatic functions were also observed, such as glycogen storage and lipid accumulation. Finally, we collected and manually curated a cell state transition resource containing 224 time-course gene expression datasets and 153 cell types. Our framework, together with the data resource, is freely available at http://pc3t.idrug.net.cn/. We believe that PC3T is a powerful tool to promote chemical-induced cell state transitions.

## Abstract (web summary)

PC3T is an in silico chemical screening pipeline to identify small-molecule treatments that induce cell state transitions for any given initial and terminal state or any intermediate state in the cell transition trajectory.

## Introduction

Cell state transition (reprogramming, differentiation and transdifferentiation) is one of the fundamental events in biology, and advances in the control and manipulation of cell identity enable the generation of desired cell types, which provide broad applications in disease modeling, drug discovery and regenerative medicine^1^. Currently, there are two main strategies to achieve cell fate conversion: (1) inducing lineage-specific transcription factors^2,3^ or (2) small-molecule stimulation^4–6^. However, the induction of exogenous transcription factors raises safe concerns for its clinical applications. In contrast, small molecules do not integrate into the genome and are highly controllable, easy to optimize, and standardize^7^, and, therefore, are promising solutions for the clinical application of cell lineage reprogramming. It is critical to screen small molecules that can induce the desired cell state transition.

Conventional phenotypic chemical screening always starts with a selected pool of compounds that target particular pathways or biological processes^8,9^. This strategy relies heavily on a priori understanding of the mechanism driving a desired cell state transition. Moreover, considering the large chemical space, the missing is inescapable because of the limited scale of the initial screening pool. Cell state transition is a dynamic process, and the identification of small molecules that promote intermediate stages in the trajectory path is important. Unfortunately, intermediate states are usually unstable and reversible, and their features are not well-characterized, which limits the use of traditional molecular screening methods.

Given that cell state transitions are associated with characteristic changes in gene expression profiles, we modeled the problem as identifying small molecules that induce similar changes and developed an in silico chemical screening pipeline, a signature-driven predictor of chemical compounds for cellular transition (PC3T). For any given initial and terminal states or any intermediate state in the cell transition trajectory, PC3T enriched candidates from among 20,768 molecules in the LINCS L1000 project database^10^ and ChemPert^11^. To validate the performance of PC3T, we applied our method to previously identified cell transitions mediated by small molecules and obtained correct predictions in most of the datasets. We then predicted and experimentally validated small molecules that convert fibroblasts into hepatic progenitor-like cells (HPLCs) and found that mouse embryonic fibroblasts (MEFs) exhibited epithelial cell-like morphology after treatment with carbidopa, LY-364747 or CHIR99021. Moreover, these molecules suppressed the expression of fibroblast-specific genes while inducing the expression of hepatocyte-specific genes. The converted cells exhibited hepatic functions, such as glycogen storage and lipid accumulation. Finally, we collected and manually curated a comprehensive time-series gene expression resource representative of the dynamic transition process and predicted the molecules inducing these changes. The datasets and online server of PC3T are freely available at http://pc3t.idrug.net.cn/. We believe that PC3T will be a valuable resource and useful server for both experimental and computational biologists who are interested in chemical-induced cell state transitions.

## Results

### Method overview

Here, we present a computational method to screen small chemical molecules that can induce desired cellular transitions. The method requires only gene expression profiles of the initial and desired cellular states. Therefore, our method can be applied to the transition between any pair of initial and query cell types, including novel cell transitions that have not been previously achieved, whether by transcription factors or chemical molecules.

**Fig. 1:**
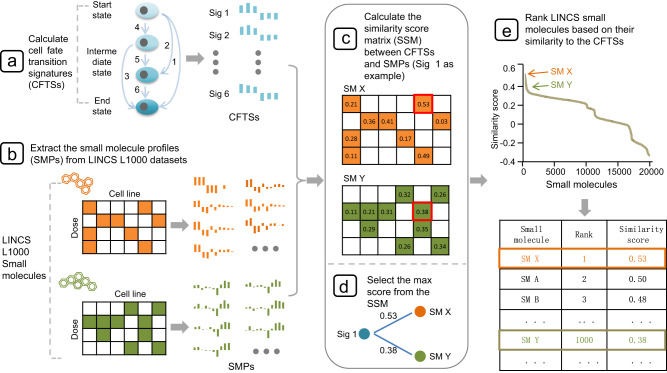
The schematic representation of the PC3T. **a** Differentially expressed genes (DEGs) as cell fate transition signatures (CFTSs) based on the expression profiles. **b** The small-molecule profiles (SMPs) were derived from the LINCS L1000 database and ChemPert. **c** The similarity score matrix between the CFTSs and SMPs was calculated via gene set enrichment analysis (GSEA). **d** The max value was used as the final similarity score for the molecule. **e** The rank of all the small molecules based on their similarity score to the CFTSs.

In the first step of the method, we identified the differentially expressed genes (DEGs) as cell fate transition signatures (CFTSs) based on the expression profiles obtained during the transition process both on bulk and single-cell levels (Fig. 1a). The small-molecule profiles (SMPs) were derived from the LINCS L1000 database and ChemPert, which houses perturbation profiles of 20,768 molecules in 20 cell lines. We averaged all of the profiles for each molecule, and the profiles of each molecule associated with different doses and cell lines were categorized independently (Fig. 1b). Then, a similarity score matrix between the CFTSs and SMPs was calculated via gene set enrichment analysis (GSEA) (Fig. 1c)^12^, and the max value was used as the final similarity score for the molecule, which was considered a measure of its reprogramming potential after optimization (Fig. 1d). Finally, we ranked all the small molecules based on their similarity score to the CFTSs, and top-ranking molecules were expected to be candidates to induce the cell state transition (Fig. 1e).

### Application to cell reprogramming from fibroblasts to iPSCs

**Fig. 2:**
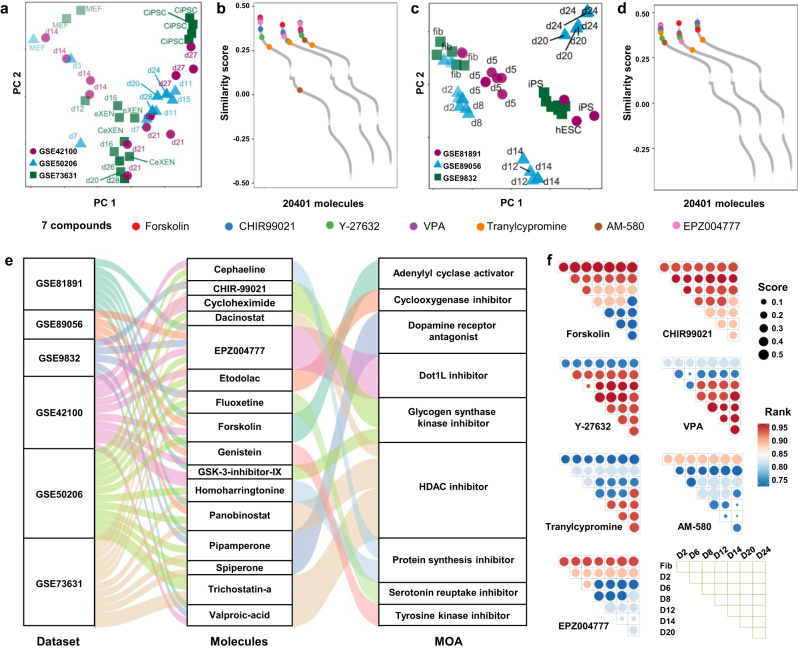
Application to cell reprogramming from fibroblasts to iPSCs in human and mouse. **a** PCA plot of three datasets of reprogrammed iPSC from fibroblasts in mouse. **b** The similarity scores for datasets in (**a**). **c** PCA plot of three datasets of reprogrammed iPSC from fibroblasts in human. **d** The similarity scores for datasets in (**c**). **e** The Sankey diagram of the associations of the datasets, top molecules and MOAs. **f** The bubble plot of the similarity score of molecules for different reprogramming stages.

Induced pluripotent stem cells (iPSCs) are an invaluable tool in regenerative medicine and are one of the most studied state transitions. In 2006, Yamanaka and colleagues induced iPSC from mouse embryonic or adult fibroblasts by introducing four factors, Oct3/4, Sox2, c-Myc, and Klf4 (OSKM)^3^. Since then, several methods have been proposed to achieve this process via either transcription factors or small molecules^5,13–15^. In this section, we applied PC3T to identify molecules that enhance the reprogramming of iPSCs, and the prediction was considered correct if the reported molecules ranked highly among all molecules. In this study, the top 5% molecules were considered as top-ranking molecules. We first selected three datasets of reprogramming of mouse iPSC from fibroblasts by transcription factors or small molecules (Fig. 2a)^6,16,17^. Multiple time points were included in these datasets (the average time point was 6.7), and the detailed information was in Supplementary Table 1. We called the cells of origin and those after transitioning as the initial cells and target cells, respectively, and calculated the similarity score of 20,401 molecules (Fig. 2b). We focused on seven molecules that had been reported to drive iPSC reprogramming, including forskolin, CHIR99021, Y-27632, VPA, tranylcypromine, AM-580 and EPZ004777. These seven molecules showed high similarity scores and ranked among the top in most of the datasets; the median ranks in the three datasets were 60,387 and 119. Target-based strategies were commonly used for molecular screening, and we compared the results obtained through PC3T with those obtained via the target-based method. A total of 3020 molecules with known targets were selected. As shown in Supplementary Fig. 1, the median ranks identified by PC3T in the three datasets were 45, 76 and 52; however, the median ranks identified by the target-based method in the three datasets were 1767, 1115 and 1575, which indicated that PC3T performed better than the target-based method.

We then applied PC3T to human reprogramming cells. Human somatic cells are refractory to chemical stimulation because of their stable epigenome^18^ and reduced plasticity;^19^ therefore, it is challenging to induce human iPSCs by chemical reprogramming. As shown in Fig. 2c, three datasets were selected^13,15,20^, and the average time point was 4.3 (Supplementary Table 1). The similarity scores of 20,401 molecules are shown in Fig. 2d. Identical results were obtained, and the median ranks of the seven molecules were 35, 159 and 105. Our results suggested that although difficult to achieve, reprograming human cells involves a similar mechanism to that identified in mouse cells, and PC3T can be used to predict molecules for both human and mouse cell reprogramming. We then compared the PC3T results with the target-based method results. As shown in Supplementary Fig. 2, the median ranks in the three datasets via PC3T were 34, 38 and 20; however, the median ranks identified by the target-based method in the three datasets were 2437, 1600 and 1756. Finally, we investigated the influence of the number of signature genes on the similarity scores. Using different numbers of signature genes (ranging from 50 to 300), we calculated the similarity scores for molecules in six datasets in three cell lines (the ASC, NPC and MCF3 cell lines). As shown in Supplementary Fig. 3a, the distribution of similarity score was influenced by signature size, therefore, we used uniform signature size in our study, and our pipeline was robust with respect to signature size, and the average correlation coefficients were higher than 0.8 when the signature size was 200 genes (Supplementary Fig. 3b). In view of the fold change of perturbation profiles (Supplementary Fig. 3c), we used the 200 most highly upregulated and downregulated genes as signatures in our study. PC3T calculated the similarity score using GSEA, which is a rank-based algorithm and performed well across datasets generated using different technologies (Supplementary Fig. 4).

In addition to the seven aforementioned molecules, we investigated other molecules with top rankings. The top 30 molecules in each of the six datasets were selected, and molecules with a known mechanism of action (MOA) were selected for further study (Supplementary Table 2). Sixteen molecules appeared in at least two datasets, as shown in Fig. 2e, among which five molecules were common to at least four datasets, including EPZ004777, forskolin, pipamperone, panobinostat and trichostatin-a. Four molecules were included in the reported cocktail, and the other 12 molecules were novel. Most of these molecules induced the up-expression of pluripotency and down-expression of fibroblast genes in the LINCS L1000 dataset (Supplementary Fig. 5). Further investigation to determine whether these molecules induce or enhance cell reprogramming of iPSCs is a worthy endeavor. Nine MOAs were involved, and the HDAC inhibitor, glycogen synthase kinase inhibitor, a Dot1L inhibitor and a Dopamine receptor antagonist were the top MOAs with most dataset-molecule pairs, indicating that these cellular pathways may play important roles during iPSC reprogramming. We found that molecules with the same MOA were inclined to be clustered together, such as cephaeline and homoharringtonine; dacinostat, panobinostat and trichostatin-a; CHIR-99021 and GSK-3-inhibitor-IX (Supplementary Fig. 6a). We further investigated the biological processes affected by these molecules using GSEA. As shown in Supplementary Fig. 6b, fibroblast-related processes were downregulated, such as fibroblast proliferation, wound healing, cell matrix adhesion, stress fiber assembly and actin filament organization. On the other hand, DNA modification and cell cycle processes were upregulated.

The gene profiles at different time points represent the transition trajectory. In addition to the origin and destination of cell transitions, PC3T can be used to predict molecules for any intermediate state pair. To illustrate this application, we used our previous dataset (GSE89056), which contained eight time points (Fig. 2c), and calculated the similarity score of the seven molecules for each time point combination. As shown in Fig. 2f, we found that the similarity scores of molecules varied by time course, which indicated that these molecules may mainly function in different transitional stages. We obtained gene signatures of different reprogramming stages^21^ and found that the fibroblast genes were downregulated by seven molecules, and other stage genes were upregulated differently (Supplementary Fig. 7a). We further calculated the mean fold change of gene signatures as signature score and found that CHIR99021 and tranylcypromine may participate in an early transition stage, while Y-27632, EPZ004777 and VPA may participate in a late transition stage (Supplementary Fig. 7b). The expression levels of VPA signatures in different time points, and the genes upregulated by VPA exhibited high expression levels at late reprogramming time points, while the genes downregulated by VPA exhibited low expression levels at late reprogramming time points (Supplementary Fig. 7c).

### Application to cell reprogramming of scRNA-seq data

**Fig. 3:**
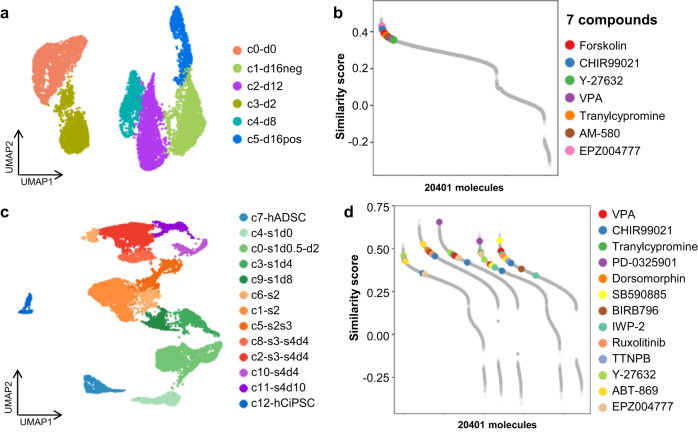
Application to cell reprogramming of single-cell data. **a** UMAP plot of the dynamic transition from fibroblasts to iPSC. **b** The similarity score of 20,401 molecules. **c** UMAP plot of integrated scRNA-seq profiles of the reprogramming early-stage samples. **d** The similarity scores of cell reprogramming using different reprogramming stages as target cells.

Recently, single-cell analysis revealed a high-resolution landscape of cell transition trajectories and helped us discover rare but important mechanisms that had been masked in bulk analysis^22–24^. Hence, single-cell analysis has become a promising tool for use in cell fate transition studies. In this section, we described our efforts to determine whether PC3T was adequate for use with single-cell datasets. We first employed our method to scRNA-seq data obtained from OSKM-induced reprogramming cells (GSE118258)^25^. A UMAP clustering of the dynamic transition from parental BJ (c0-d0) to D16+ cells (c5-d16pos) is shown in Fig. 3a. Using markers (differentially expressed genes) in parental BJ and D16+ cells as the required signature genes, we calculated the similarity scores between molecular perturbation profiles and these signature genes. As shown in Fig. 3b, the seven molecules showed high similarity scores (ranging from 0.43 to 0.34) and rankings (ranging from 63 to 1479, median value is 620) among all the 20,401 molecules. Recently, the Deng group reprogrammed human fibroblasts into hCiPS cells via small molecules. The whole procedure was categorized into four stages, and 26 molecules were involved in the process^4^. As shown in Fig. 3c, all the cells were clustered into 13 groups (GSE178325). We used human adult adipose-derived mesenchymal stromal cells (hADSCs) as the initial cells and clusters of the end time point of each stage and hCiPSC as the target cells (c0-s1d0.5-d2, c1-s2, c2-s3-s4d4, c11-s4d10 and c12-hCiPSC); we thus calculated similarity scores of the LINCS molecules (Fig. 3d). Thirteen of the 26 molecules were found in LINCS data, and the median rank of the molecules involved at a specific stage were 529, 2905, 3670, 1946 and 1605 respectively. In particular, tranylcypromine (TPCA-1 inhibitor), SB590885 (B-Raf inhibitor) and PD-0325901 (MEK inhibitor) showed high similarity scores in different stages; however, their rank was lower than the example above, which may have been a result of many more molecules included in the chemical cocktail. In summary, our method can be used for molecular screening based on scRNA-seq data of cell state transition.

### Application to cell transition into neurons

**Fig. 4:**
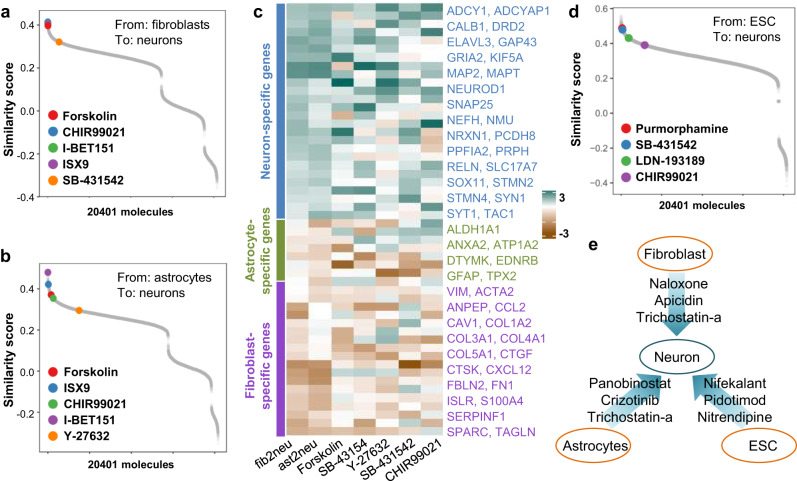
Application to cell transition into neurons. **a**, **b** The similarity scores for cell transition from fibroblasts to neurons (**a**) and from astrocytes to neurons (**b**). **c** Heatmap showing the expression of fibroblast-, astrocyte- and neuron-specific genes during cell transitions and molecule treatment. **d** The similarity scores of cell differentiation from ESC to neurons. **e** Top molecules for three cell transitions.

Because of its limited regenerative ability, the mammalian central nervous system is a desirable target for assessing in vivo chemical reprogramming. It has been reported that different cell types can be converted into functional neurons^26–28^. In this section, we first applied PC3T to predict the chemical molecules that can reprogram fibroblasts and astrocytes into neurons directly. Li et al. developed a cocktail of small molecules that direct drove lineage reprogramming of mouse fibroblasts into functional neurons^27^. We used fibroblasts and the cells induced 19 days as initial cells and target cells respectively (GSE68715), and calculated the similarity score between the transition signatures and LINCS perturbation profiles. We focused on the similarity score of the FICB (forskolin, ISX9, CHIR99021 and IBET151) cocktail. As shown in Fig. 4a, these four molecules yielded high similarity scores (0.397, 0.414, 0.407 and 0.405, respectively) and top rankings (25, 9, 13 and 15) from among all 20401 LINCS molecules. We also noticed another molecule, SB431542, which enhanced the survival and neurite outgrowth of induced neurons but was dispensable for generating neuron generation. The similarity score of SB431542 was 0.321, and it ranked 1366 among all 20,401 molecules. Astrocytes are ideal targets for in vivo reprogramming because they are among the major cell types that respond, proliferate, and assemble to enclose necrotic lesions. The Deng group reprogrammed astrocytes into neurons using a chemically defined cocktail called DFICBY (DBcAMP, forskolin, ISX9, CHIR99021, IBET151, and Y-27632)^26^. We calculated the similarity score between the transition signatures (GSE164421) and molecular perturbation profiles. Five molecules in DFICBY were included in the LINCS dataset except for DBcAMP, and four of these five molecules (forskolin, ISX9, CHIR99021 and IBET151) exhibited high similarity scores (0.371, 0.422, 0.355 and 0.480) and top rankings (385, 58, 620 and 2) among all 20401 LINCS molecules, while the similarity score and rank of Y-27632 were 0.295 and 3740 respectively (Fig. 4b). As shown in Fig. 4c, fibroblast-specific genes and astrocyte-specific genes were downregulated during both the reprogramming procedure and molecular treatment, and neuron-specific genes were upregulated. Pluripotent stem cells are promising sources of cells for application in regenerative medicine. We applied PC3T to predict small molecules that lead to the directed differentiation of embryonic stem cells into neurons (GSE32658). Three molecules (purmorphamine, SB-431542, and LDN-193189) used by Sonja et al.^28^ exhibited high similarity scores (0.488, 0.479 and 0.431) and top rankings (122,169 and 923) (Fig. 4d). Collectively, our method consistently predicted chemical molecules that induced the transition of different cell types into neuron. Finally, we investigated the top-ranked molecules that were not included in the reported cocktail for each of the reprogramming procedures (Fig. 4e). Three HDAC inhibitors, panobinostat, trichostatin-a and apicidin, were enriched, and panobinostat and trichostatin-a were also predicted to be candidates for fibroblast to iPSC reprogramming (Fig. 2e). The potential role played by these molecules is worthy of further investigation.

### Prediction and validation of small molecules inducing mouse fibroblast transdifferentiation into hepatic progenitor-like cells

**Fig. 5:**
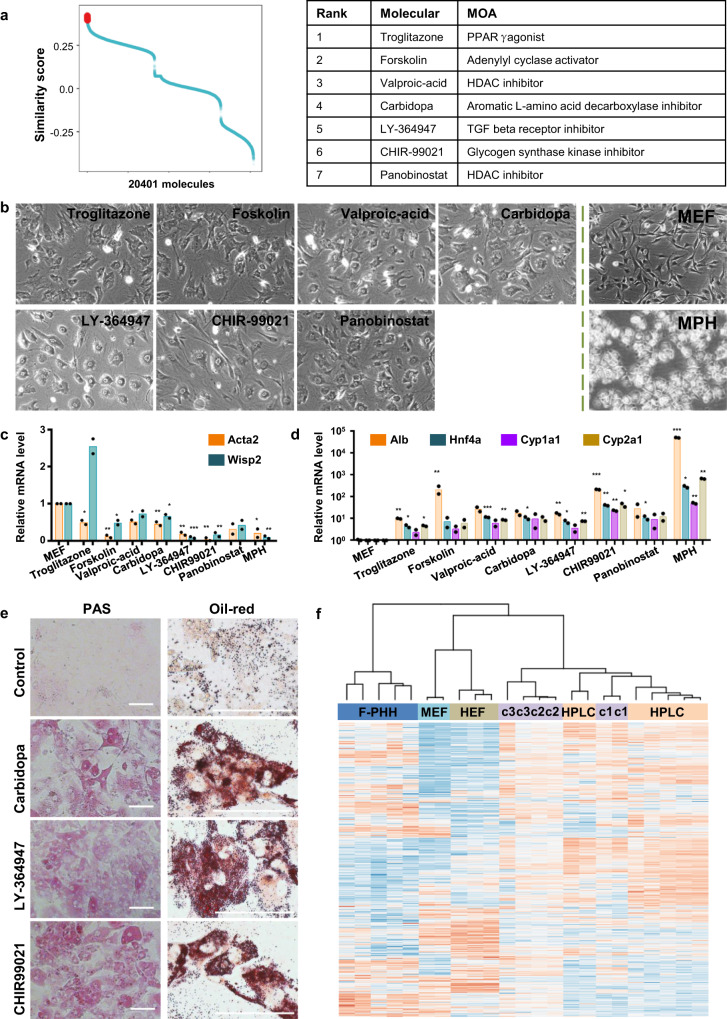
The prediction and validation of small molecules inducing mouse fibroblasts into hepatocytes. **a** The similarity scores (left) and top molecules (right) of cell transition from fibroblasts to hepatocytes. **b** Morphology of cells treated by seven molecules (left) and MEF and mouse primary hepatocyte (MPH) (right). Scale bar, 50 µM. **c**, **d** The expression of fibroblast-specific genes (**c**) and hepatocyte-specific genes (**d**) after molecules treatment (10 µM). There were two biologically independent samples. Statistical significance was determined with a two-tailed unpaired Student’s *t*-test, and the estimated effect size was determined using Cohen’s D (Supplementary Tables 4 and 5). Asterisks indicate *p*-values: *p < 0.05; **p < 0.01; ***p < 0.001. **e** Hepatic functions in converted cells: Oil Red O staining and PAS staining. Scale bar, 50 µM. **f** Hierarchical clustering of global gene expression of MEFs, converted cells treated by three molecules (c1: CHIR-99021, c2: LY-364947; c3: carbidopa), and HEF, F-PHH and HPLC from Deng group.

In the previous sections, we applied our method to the cell station transitions that have been previously achieved via chemical induction to illustrate the performance of PC3T, which produced results consistent with the experimental data. In this section, we employed PC3T to screen molecules that induce the reprogramming of fibroblasts into hepatocytes. The liver is a pivotal organ for regulating many physiological processes, and the generation of surrogate hepatocytes is important to cell-based approaches in liver disease treatment and drug pharmacokinetics analysis^29^. The Hui group directly reprogrammed fibroblasts into functional and expandable hepatocytes both in mouse and human by introducing lineage-specific transcription factors^30,31^. The Deng group developed a two-step lineage reprogramming strategy by mimicking a natural regeneration route. Specifically, fibroblasts were first reprogrammed by hepatic transcription factors into proliferative human hepatic progenitor-like cells (hHPLCs), and then, the hHPLCs were chemically induced to become functionally competent hepatocytes^32^. In our study, using fibroblasts and hHPLCs as the source cells and target cells, respectively, we screened candidate molecules by PC3T. The similarity scores of all 20,401 molecules are shown in Fig. 5a, and the seven top molecules were chosen for experimental validation, including troglitazone, forskolin, valproic-acid, carbidopa, LY-364947, panobinostat and CHIR-99021 (Supplementary Table 3). We first investigated morphology changes induced by molecular treatment. As shown in Fig. 5b, MEFs treated with LY-364947, CHIR-99021, carbidopa or VPA displayed epithelial cell-like morphology. The expression of fibroblast genes such as Acta2 and Wisp2 was decreased in cells treated with these molecules, particularly forskolin, VPA, carbidopa, LY-364947 and CHIR-99021 (Fig. 5c, Supplementary Table 4). On the other hand, the expression of genes specific to hepatocytes, such as Alb, Hnf4α, Cyp1a1 and Cyp2a1, was increased in the treated cells (Fig. 5d, Supplementary Table 5). It was reported that hHPLC-derived resembled freshly isolated primary hepatocytes (F-PHHs) in cell identity and functionality hepatocytes^32^. Five of the seven molecules also derived the top-ranking (top 5%) using F-PHHs as target cells, including forskolin, carbidopa, LY-364947, panobinostat and CHIR-99021. According to the morphology and gene expression results, we chose three molecules for further study. The converted cells displayed hallmark hepatic functions, such as accumulation of fat droplets and glycogen synthesis (Fig. 5e). Hierarchical clustering revealed that converted cells clustered closely with HPLCs, but were distinct from MEFs and human embryonic fibroblasts (HEFs) (Fig. 5f). We then reduced the concentration of the molecular treatments by one-half (5 uM) and treated the MEFs. The changes we observed were consistent with the aforementioned changes in the expression of fibroblast and hepatocyte genes (Supplementary Fig. 8, Supplementary Tables 6 and 7). The robust performance of PC3T confirmed combining and optimizing these molecules, which is worthy of further investigation.

### Construction of resource and webserver

**Fig. 6:**
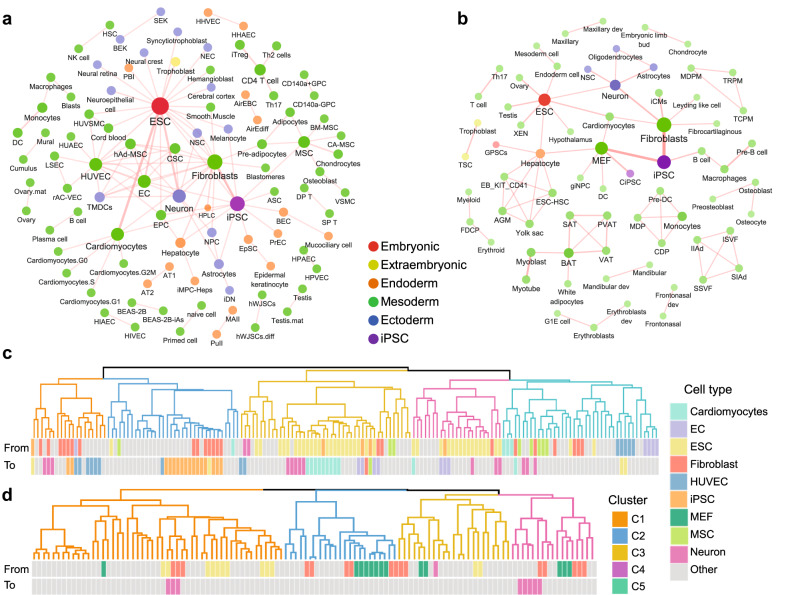
The construction of resource and webserver of PC3T. **a**, **b** The landscape of cell state transitions in human (**a**) and mouse (**b**). **c**, **d** The classification of cell state transitions according to the similarity of their signatures in human (**c**) and mouse (**d**).

We previously presented an online resource with the time-course gene expression data during cell state transitions in bulk tissues of human and mouse^33^. With the rapid advances, an increase in successful cell fate reprogramming has been achieved in recent years. Moreover, public resources have accumulated large amounts of gene expression data, especially scRNA-seq data, characterizing the dynamic transition process. Herein, we utilized text mining to collect public datasets and manually curated them to provide concise experimental descriptions and annotations of the key transition time points for every sample in these datasets. In total, 224 datasets were collected, 132 with human data and 92 with mouse data (Table 1), and 153 cell types were included. The average time points were 5.18, 4.71 for human and 5.95 for mouse. These data offer a comprehensive roadmap to describe diverse cell state transitions. The landscapes of the cell state transitions in human and mouse deposited in PC3T was shown in Fig. 6a, b (Supplementary Table 8). Hence, the differentiation of embryonic stem cells and reprogramming of fibroblast into iPSCs have been the most widely studied transitions. Moreover, cell state transitions can be classified into different groups according to the similarity of their expression signatures (Fig. 6c, d). The upregulated and downregulated genes common to the same clusters are shown in Supplementary Figs. 9 and 10.

**Table 1:** Statistics of cell state transition datasets deposited in PC3T.

Species	Datasets	Cell types	Average time points
Human	132	98	4.71
Mouse	92	72	5.95
Total	224	153	5.18

In the previous sections, we confirmed the performance of PC3T using both computational and experimental results. To facilitate the application of PC3T, we constructed a webserver, which is freely available at http://pc3t.idrug.net.cn/. The online tool comprises LINCS perturbation gene profiles and cell fate reprogramming datasets collected to date. The powerful and user-friendly interface enables experimental researchers to predict and visualize the molecules for the given cell state transition in a flexible and diverse manner. PC3T also supports the query and download of gene expression profiles and signatures for a specific cell transition process. Finally, users are allowed to upload their custom time-series gene expression data for analysis.

## Discussion

Cell state transition has been a rapidly advancing field in recent decades^2,3,9,26,27,30^, and small molecules provide a temporal and highly tunable approach for the clinical application of cell reprogramming. Scalable throughput chemical screening has become an urgent challenge, which is currently limited by the cost of exhaustive experimental testing of plausible sets of molecules, and in silico methods are urgently needed. Inspired by the connectivity map (CMap) concept^34,35^, we used the changes in gene expression profiles as indicators reflecting the underlying mechanisms of cell state transitions and developed PC3T, an unbiased molecular screening for cell transitions. PC3T used the LINCS chemical pool, which is more comprehensive than the conventional phenotype-based chemical pool used for screening. Moreover, PC3T does not rely on expert knowledge of lineage-specific genes or pathways.

To illustrate the performance of PC3T, we applied our method to cell state transitions that have been achieved via small-molecule treatment. Three main types of transitions were used to evaluate PC3T: cellular reprogramming^6,13,15–17,20^, differentiation^28^ and transdifferentiation^26,27^. The results showed that our method consistently enriched small molecules that had been experimentally validated regardless of the induction condition. Moreover, PC3T performed well with scRNA-seq data of chemical-induced reprogramming^4,25^. Focusing on the dynamic intermediate states also distinguishes PC3T from similar methods^36^.

We further predicted small molecules that reprogrammed MEFs into HPLCs. Four molecules that induced changes in the morphology and expression of fibroblast genes and hepatocyte genes were enriched: carbidopa, CHIR-99021 and LY-364947. MEFs treated by these molecules exhibited HPLC-like gene expression profiles and hepatic functions. CHIR-99021 has been reported to reprogram fibroblasts into iPSCs and other cell types^37^. LY-364947 is a TGF-beta 1 receptor inhibitor, and the anti-fibrotic effect of LY-364947 has been previously reported in silicosis treatment^38^, proliferative vitreoretinopathy prevention^39^, and central nervous system injury^40^. Carbidopa is an aromatic-L-amino-acid decarboxylase inhibitor. It is used in Parkinson’s disease to reduce peripheral adverse effects of levodopa^41^. Their roles in cell fate reprogramming are worthy of further investigation.

With the rapid development of cell fate reprogramming, a large amount of datasets have been accumulated, with single-cell data in particular. We therefore collected and manually curated 224 time-course gene expression datasets during cell state transition, including 153 cell types, and the resource is freely available in our PC3T. These datasets not only provide valuable resources to characterize the complicated transition trajectory path but also suggest the barriers that must be overcome during reprogramming^25^.

There are several limitations that need to be further addressed. First, our screening pool was limited to molecules for which transcriptional profiles had already been experimentally assessed. Fortunately, cost-effective sequencing methods have been developed, such as DRUG-seq^42^ and sci-Plex^43^, which enable researchers to derive massive chemical transcriptomics at a very low cost. In addition, machine learning algorithms have been proposed to generate perturbation transcriptional profiles relying only on chemical formulas^44^. These experimental and computational methods will largely increase the utility of our approach. In addition, DEGs were used as signatures in our current pipeline, and other algorithms identifying driver regulators of cell fate decisions, especially with single-cell data^45,46^, can be integrated as options in further iterations of PC3T.

In summary, this study presented an in silico screening framework to enrich small molecules that induce cell state transitions, and these molecules could be promising candidates to induce and enhance cell transition. We believe that PC3T will be a powerful server and resource to promote chemical-induced reprogramming.

## Methods

### Bulk data

P3CT first identified the change in gene expression between the required cell transition states. For bulk tissue data, the processed series matrix file was retrieved from the GEO, and the probe IDs were converted to Refseq IDs with Brainarray Chip Description Files (CDFs). We identified the fold change required using the limma R package and converted the gene IDs into Entrez IDs using the clusterProfiler R package^47^.

### Single-cell data

For scRNA-seq data, the preliminary filtered data generated from Cell Ranger were used for downstream analysis. Single-cell data were processed for dimension reduction and unsupervised clustering by following the workflow in Seurat^48^. In brief, 2000 highly variable genes were selected by using Seurat “FindVariableGenes” function. Then, the principal component analysis (PCA) matrix with 30 components was calculated to perform clustering and uniform manifold approximation and projection (UMAP) dimensionality reduction. All of the cells were clustered using the “FindClusters” function with a resolution of 0.2. We used the “FindAllMarkers” function based on normalized data to identify DEGs, and the *p*-value was adjusted using Bonferroni correction based on the total number of genes in the dataset.

Finally, the homologs gene mapping between human and mouse was conducted using biomaRt R package^49^.

### Molecular perturbation gene profiles

The L1000 data were obtained from the Library of Integrated Network-based Cellular Signatures (LINCS) project^10^. The LINCS perturbation response transcriptional profiles were generated using the L1000 assay, which is a high-throughput bead-based assay that measures the expression of 978 representative landmark transcripts^10^. Level 4 plate-normalized data in the March 2017 datasets were downloaded from the LINCS Data Portal [http://lincsportal.ccs.miami.edu/datasets/#/view/LDS-1372]. An additional preprocessing step was performed for all gct and gctx files by using the “parse.gctx” function in the “cmapR” R package. Gene expression profiles were aggregated for samples on the basis of molecule and cell line (technical/biological replicates of the small molecule). We also integrated transcriptional profiles in response to perturbations across non-cancer cell types from ChemPert^11^.

### GSEA

The similarity score was calculated using gene set enrichment analysis (GSEA), which was initially proposed by Lamb et al.^34^. Briefly, a nonparametric, rank-based pattern-matching strategy based on the Kolmogorov-Smirnov (KS) statistic was used to assess the enrichment of disease genes in a ranked drug gene expression list^12^. The “fgsea” package was employed to calculate the similarity score, which ranged from −1 to 1. A high positive score indicates an obverse relationship between the cell transition and the molecular treatment, while a low negative score indicates a reverse relationship between them. The enrichment score of upregulated signature (ES_up_) and downregulated signature (ES_dn_) were calculated respectively, and the final similarity score was calculated using the formula 1 as below:

1 $${{{{{\rm{similarity\; score}}}}}}=\frac{{{ES}}_{{up}}-{{ES}}_{{dn}}}{2}$$

### Target-based score

The target-based method is commonly used in drug development. In this study, we assumed that when the expression of a gene was downregulated during the cell transition, the molecules targeting this gene induced a cell transition. A total of 3020 molecules with known targets were selected from the LINCS datasets. For a given cell transition, we first ranked the gene list according to the fold change (from highest to lowest) and then calculated the target-based score of a molecule for the given cell transition using the formula 2 as below:

2 $${{{{{\rm{target}}}}}}-{{{{{\rm{based\; score}}}}}}=\frac{\frac{1}{{{{{{\rm{M}}}}}}}{\sum }_{1}^{{{{{{\rm{M}}}}}}}{{{{{{\rm{R}}}}}}}_{{{{{{\rm{m}}}}}}}}{{{{{{{\rm{N}}}}}}}_{{{{{{\rm{cell}}}}}}}}$$

where M is the number of targets of the molecular, R_i_ is the rank of target m in the ranked gene list, and N_cell_ is the length of the ranked gene list.

### Isolation and culture of Mouse embryonic fibroblasts and mouse primary hepatocytes

MEFs were isolated from E13.5 embryos of a C57BL/6J mouse (GemPharmatech Co. Ltd. Nanjing). The head, tail, limbs and internal organs were removed, and the rest tissues were cut into pieces and digested with 0.05% trypsin into single-cell suspensions. MEFs were cultured in DMEM plus 10% FBS (Bioind) and 100 units/ml penicillin as well as 100 μg/ml streptomycin (Gibco) at 37 °C with 5% CO_2_. The passage 3 to passage 5 of MEFs were used for the examination of chemicals effect. MPHs from C57BL/6J mice were isolated according to a previous protocol^50^. In brief, MPHs were isolated using the collagenase IV (1 mg/ml, Worthington) perfusion method. Then the cell suspension was filtered by a 70-μm cell strainer (Falcon) to obtain a single-cell suspension. Blood cells and dead cells as well as cell debris were discarded through centrifuge at 50 × g for 2 min. The isolated primary MPHs were then plated into tissue culture dishes coated by rat tail collagen and cultured in DMEM plus 10%FBS for 4 h. The medium was replaced by DMEM plus N2/B27 supplement (Gibco, N2 supplement, 17502-048; B27 supplement, 17504044) for MPHs maintenance.

### The experiment procedure of MEFs treated by molecules

For this, 0.5 × 10^6^ P3-P5 MEFs were plated onto 6 cm cell culture dishes and cultured in DMEM supplemented with 10% FBS, 100 units/ml penicillin, and 100 μg/ml streptomycin (Gibco) at 37 °C with 5% CO_2_ for 2 h for attachment. After 2 h, molecule was added to the culture medium at dosages of 5 and 10 μM to treat the MEFs. The culture medium was changed every 3 days while the molecule was continuing to treat the MEFs. The PAS staining system was purchased from Sigma-Aldrich. Cultures were fixed with 4% paraformaldehyde (DingGuo) and stained according to the manufacturer’s instructions. Lipid detection was performed with a Lipid (Oil Red O) Staining Kit (Sigma) according to the manufacturer’s instructions.

### RNA sequencing and bioinformatics analysis

We performed RNA-seq for MEFs, and cells treated by carbidopa, LY-364947 and CHIR-99021. Total RNA was isolated using the RNeasyMini kit (QIAGEN). RNA sequencing libraries were prepared using the NEBNext UltraTM RNA Library Prep kit for Illumina (NEB, USA) following the manufacturer’s recommendations. The fragmented and randomly primed 150-bp paired-end libraries were sequenced on Illumina Novaseq 6000 platform. The generated sequencing reads were mapped against the human genome build mm10 using STAR(v2.4.2a)^51^, and the read counts for each gene were calculated using featureCounts. Gene expression was normalized by DESeq2. Unsupervised hierarchical clustering of RNA-seq data was conducted by the hclust package in R (R 3.4.3). The RNA sequencing data are available in the Gene Expression Omnibus (GEO) under the accession number GSE231967.

### Real-time PCR assay

The seven small-molecule compounds were purchased from Topscience (China); the CAS ID and product ID are in Supplementary Table 3. MEFs were treated with chemicals for 7 days, and total RNA was isolated using the RNeasy Micro Kit (QIAGEN). RNA was converted to cDNA using First-Strand Synthesis SuperMix for quantitative real-time PCR (qRT-PCR) (INVITROGEN). PCR was carried out using Power SYBR Green PCR Kit (Applied Biosystems, Foster City, CA) and a LightCycler 96 Real-Time PCR System (Roche, Mannheim, Germany). The data were analyzed using the 2^−ΔΔCt^ method. The primers were listed in Supplementary Table 9.

### Statistics and reproducibility

All statistical tests used, sample sizes, and the number of replicates are described in the corresponding methods.

### Reporting summary

Further information on research design is available in the Nature Portfolio Reporting Summary linked to this article.

### Supplementary information

Supplementary Information

Reporting Summary

## Data Availability

The raw data and the processed data in the RNA-seq analysis were deposited in the GEO (GSE231967). Source data for the graphs in the main figures are available as supplementary data, and any remaining information can be obtained from the corresponding author upon reasonable request.

## Code availability

Our framework, together with the data resource, is freely available at http://pc3t.idrug.net.cn/.
