# Supplementary material for: PC3T: a signature-driven predictor of chemical compounds for cellular transition
Source: Commun Biol. 2023 Sep 27;6:989. doi: 10.1038/s42003-023-05225-y (PMC10533498; doi:10.1038/s42003-023-05225-y)
Supplement: Supplementary file 1 — Supplementary Information [file 42003_2023_5225_MOESM1_ESM.pdf]

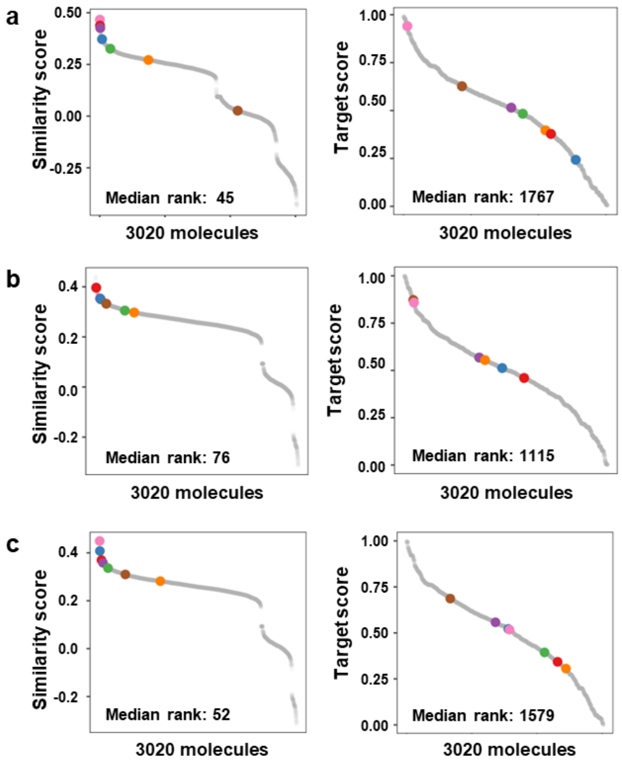

Supplementary Figure 1. The similarity scores (left) and target scores (right) of 3020 molecules in 3 mouse reprogramming datasets (a: GSE42100; b: GSE50206; c: GSE73631).

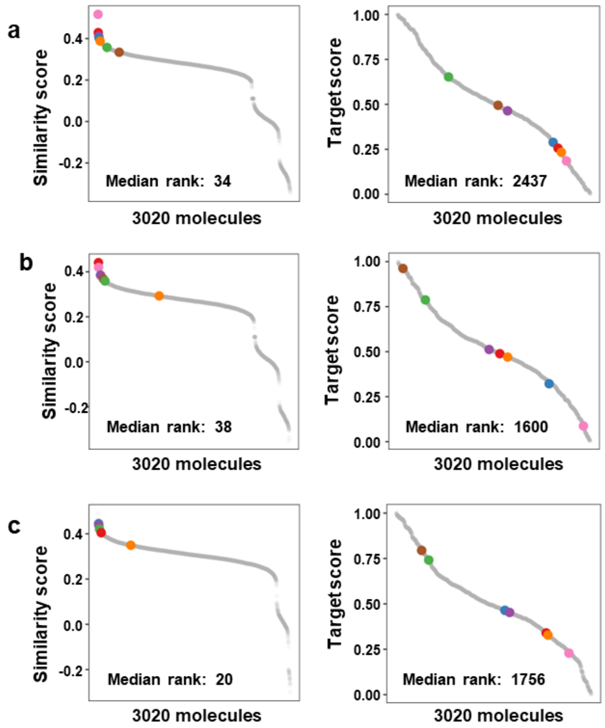

Supplementary Figure 2. The similarity scores (left) and target scores (right) of 3020 molecules in 3 human reprogramming datasets (a: GSE81891; b: GSE89056; c: GSE9832).

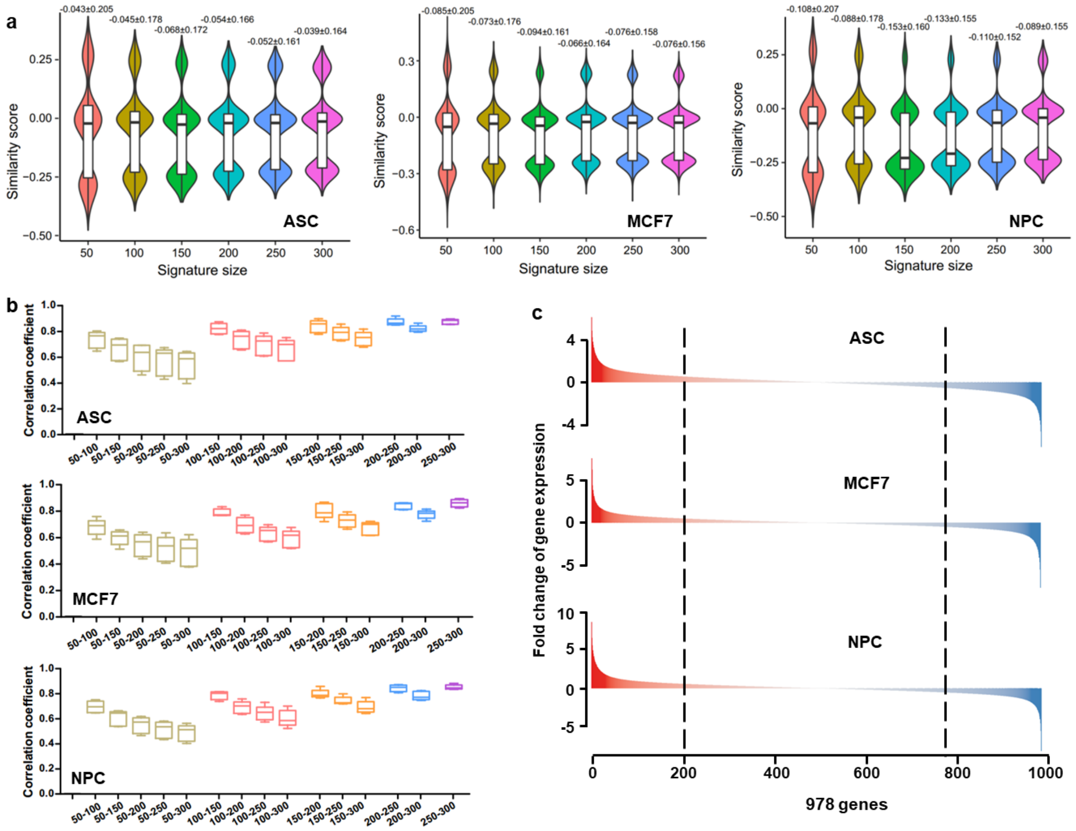

Supplementary Figure 3. The influence of the signature genes size. (a) The distribution of similarity score using different numbers of signature genes in three cell lines. (b) The correlation coefficient of similarity scores using different signature size (range from 50 to 300) in three cell lines. (c) The fold change of 978 genes in three cell lines in LINCS.

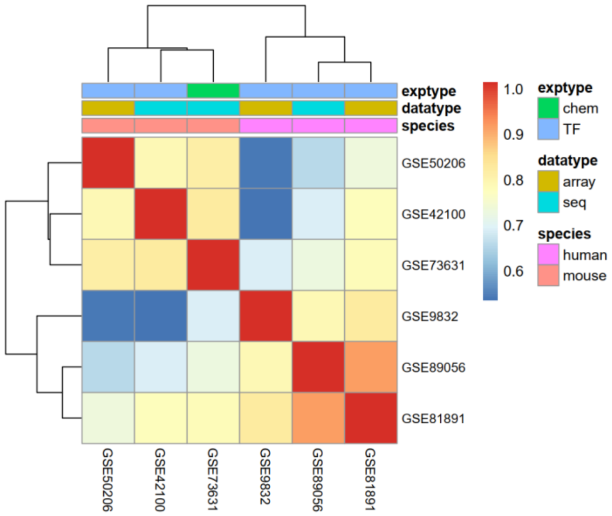

Supplementary Figure 4. The correlation between results of datasets in different species (mouse, human), using different technologies and employing different reprogramming techniques.

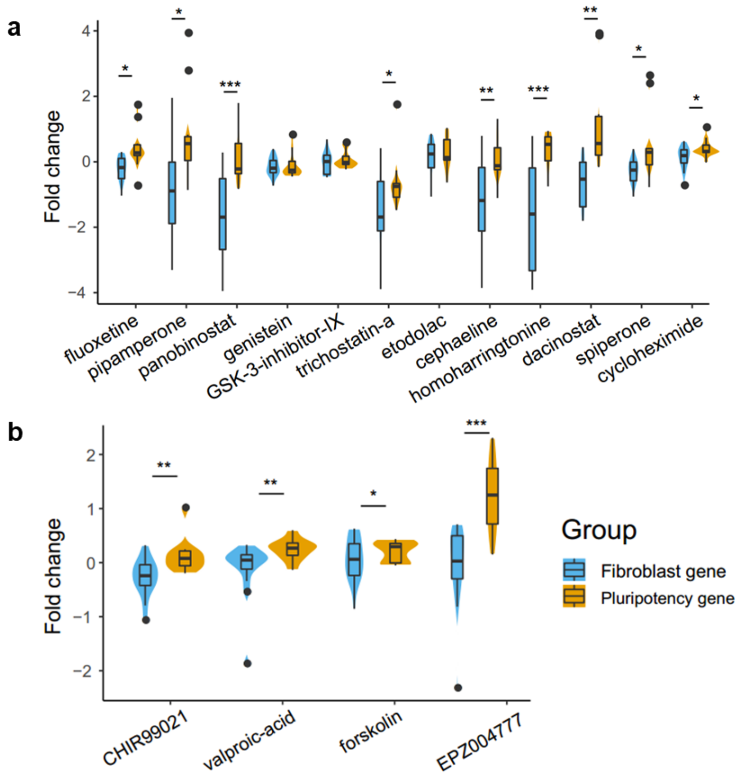

Supplementary Figure 5. The fold change of fibroblast genes and pluripotency genes in LINCS L1000 dataset after treated by 12 novel molecules (a) and 4 reported molecules (b).

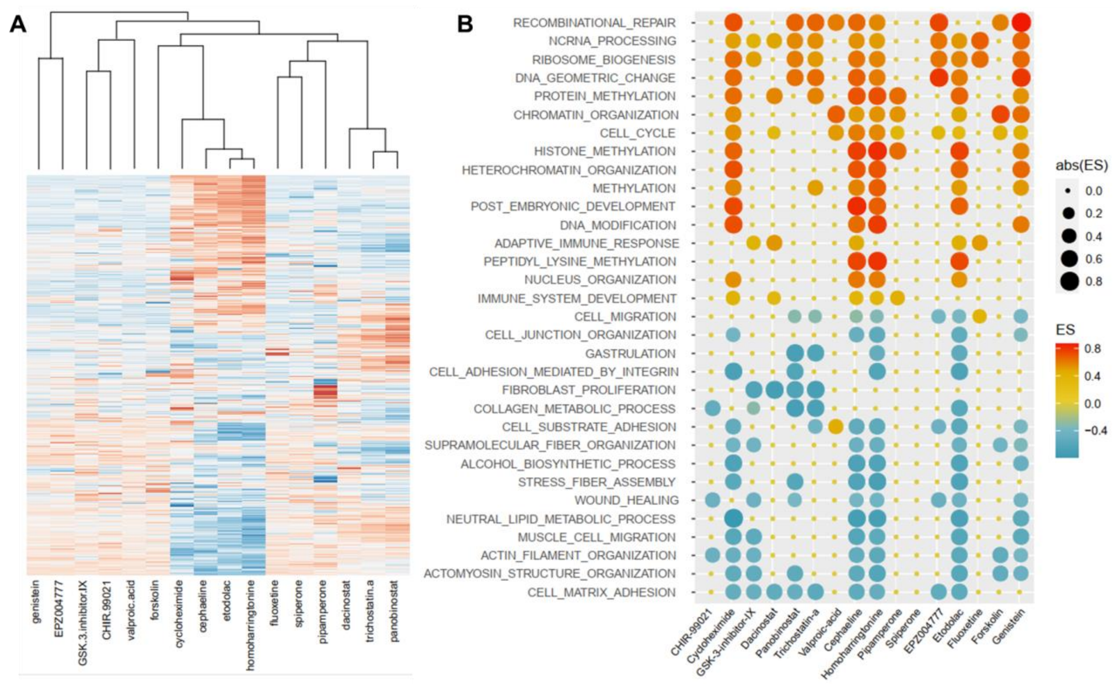

Supplementary Figure 6. The genes (a) and biological processes (b) affected by 16 molecules.

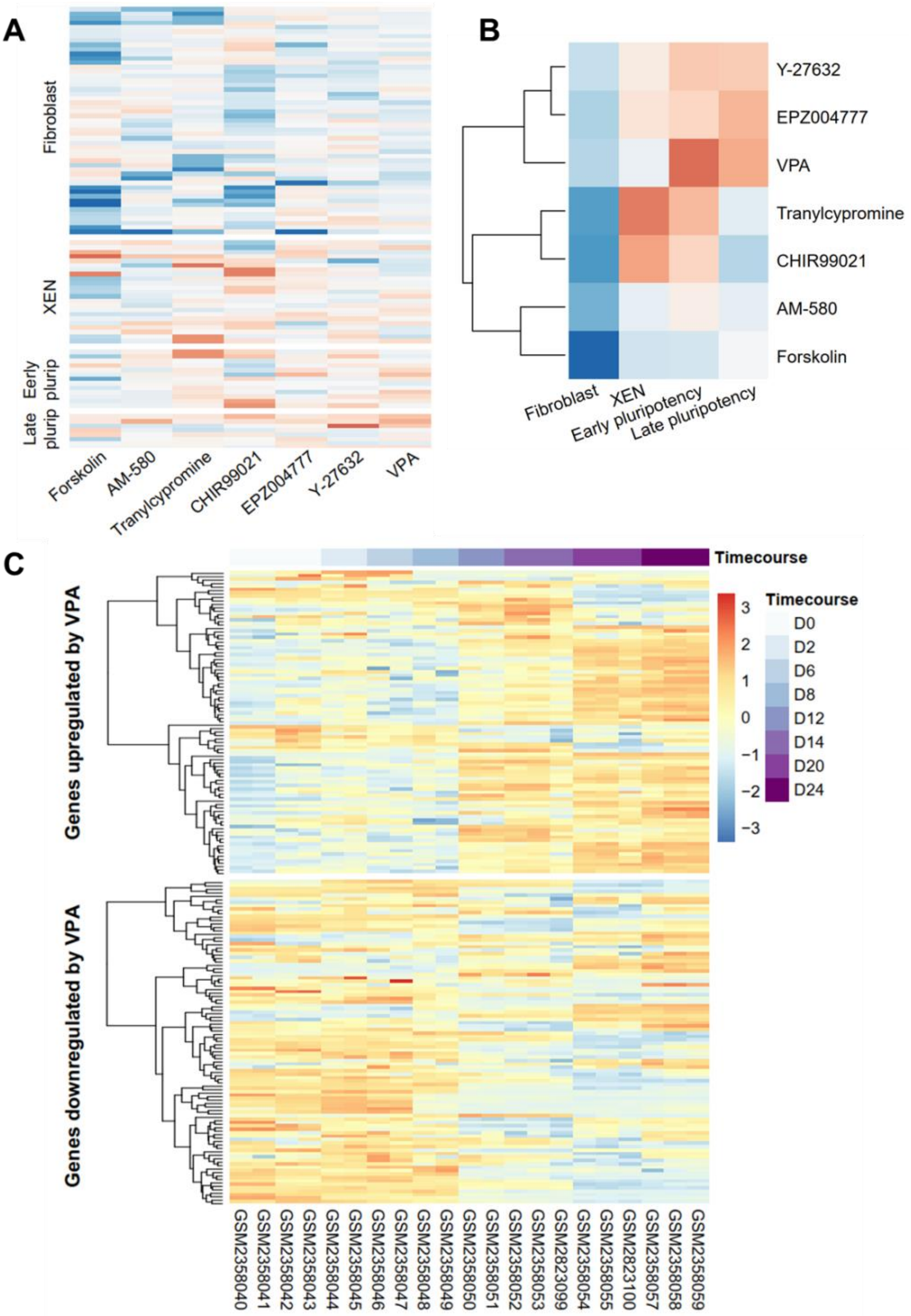

Supplementary Figure 7. The molecule scoring during reprogramming timecourse. (a) The expression change of reprogramming stage-specific gene signatures induced by 7 molecules. (b) The signature score of 7 molecules. (c) The expression of upregulated and downregulated genes by VPA during reprogramming timecourse.

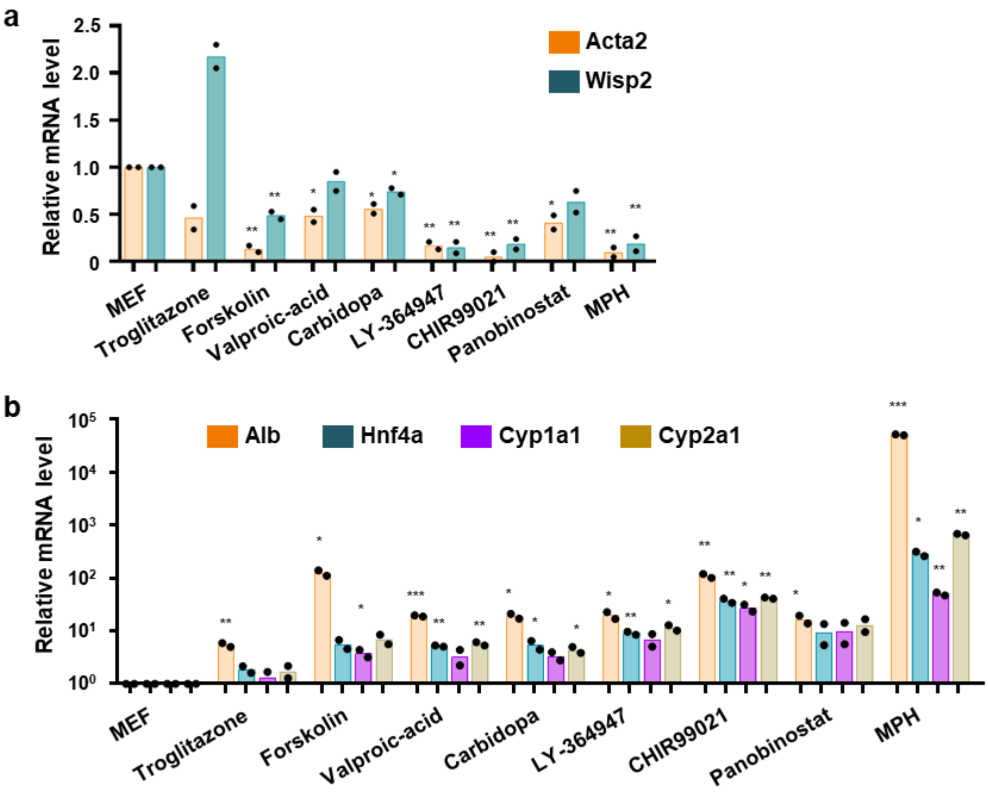

Supplementary Figure 8. The expression of fibroblast-specific genes (a) and hepatocyte-specific genes (b) after molecules treatment (5 uM). There were 2 biologically independent samples. Statistical significance was determined with two-tailed unpaired Student's t-test, and the estimated effect size was determined using Cohen's D (Supplementary Table 6 and 7). Asterisks indicate p values: *$p < 0.05$; **$p < 0.01$; ***$p < 0.001$.

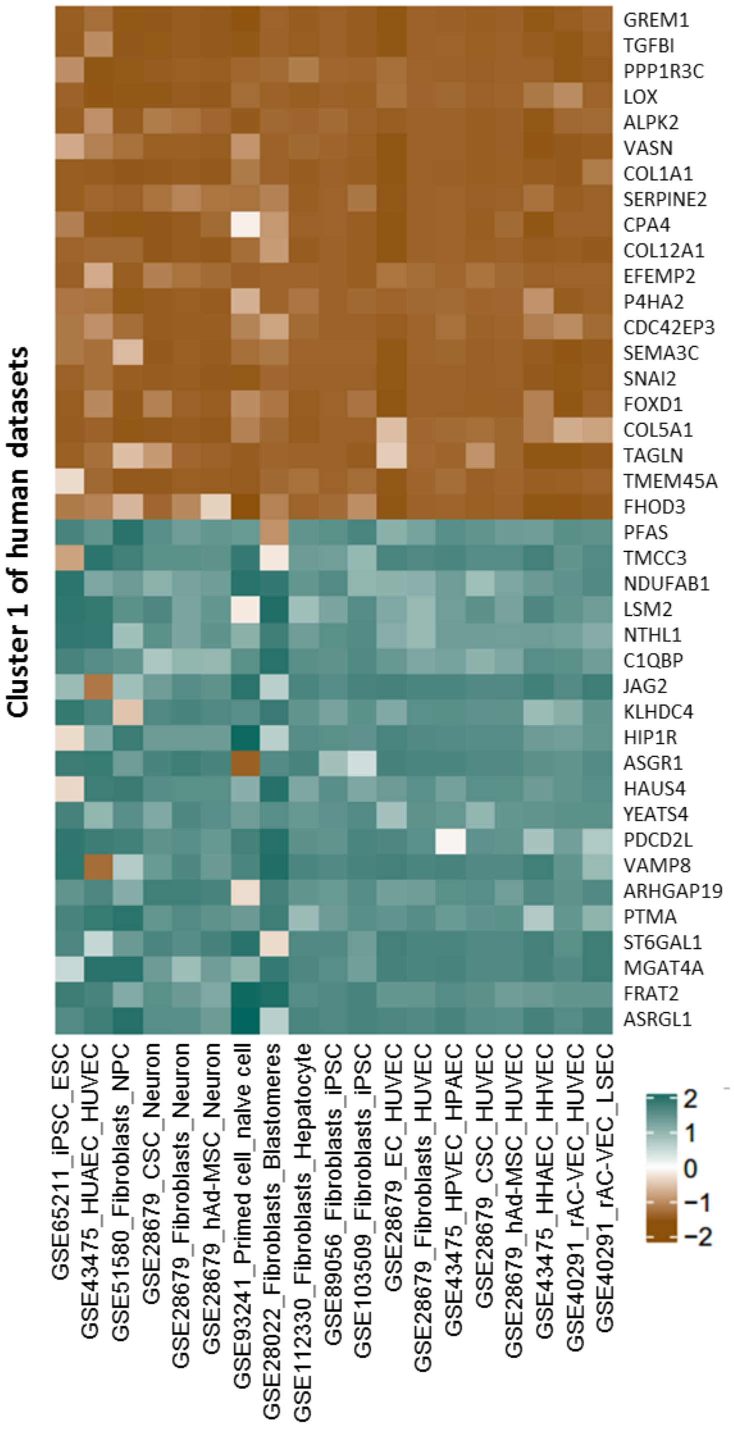
Cluster 1 of human datasets
GREM1
TGFBI
PPP1R3C
LOX
ALPK2
VASN
COL1A1
SERPINE2
CPA4
COL12A1
EFEMP2
P4HA2
CDC42EP3
SEMA3C
SNAI2
FOXD1
COL5A1
TAGLN
TMEM45A
FHOD3
PFAS
TMCC3
NDUFAB1
LSM2
NTHL1
C1QBP
JAG2
KLHDC4
HIP1R
ASGR1
HAUS4
YEATS4
PDCD2L
VAMP8
ARHGAP19
PTMA
ST6GAL1
MGAT4A
FRAT2
ASRGL1
GSE65211_iPSC_ESC
GSE43475_HUAEC_HUVEC
GSE51580_Fibroblasts_NPC
GSE28679_CSC_Neuron
GSE28679_Fibroblasts_Neuron
GSE28679_hAd-MSC_Neuron
GSE93241_Primed cell_naïve cell
GSE28022_Fibroblasts_Blastomeres
GSE112330_Fibroblasts_Hepatocyte
GSE89056_Fibroblasts_iPSC
GSE103509_Fibroblasts_iPSC
GSE28679_EC_HUVEC
GSE28679_Fibroblasts_HUVEC
GSE43475_HPVEC_HPAEC
GSE28679_CSC_HUVEC
GSE28679_hAd-MSC_HUVEC
GSE43475_HHAEC_HHVEC
GSE40291_rAC-VEC_HUVEC
GSE40291_rAC-VEC_LSEC
2
1
0
−1
−2

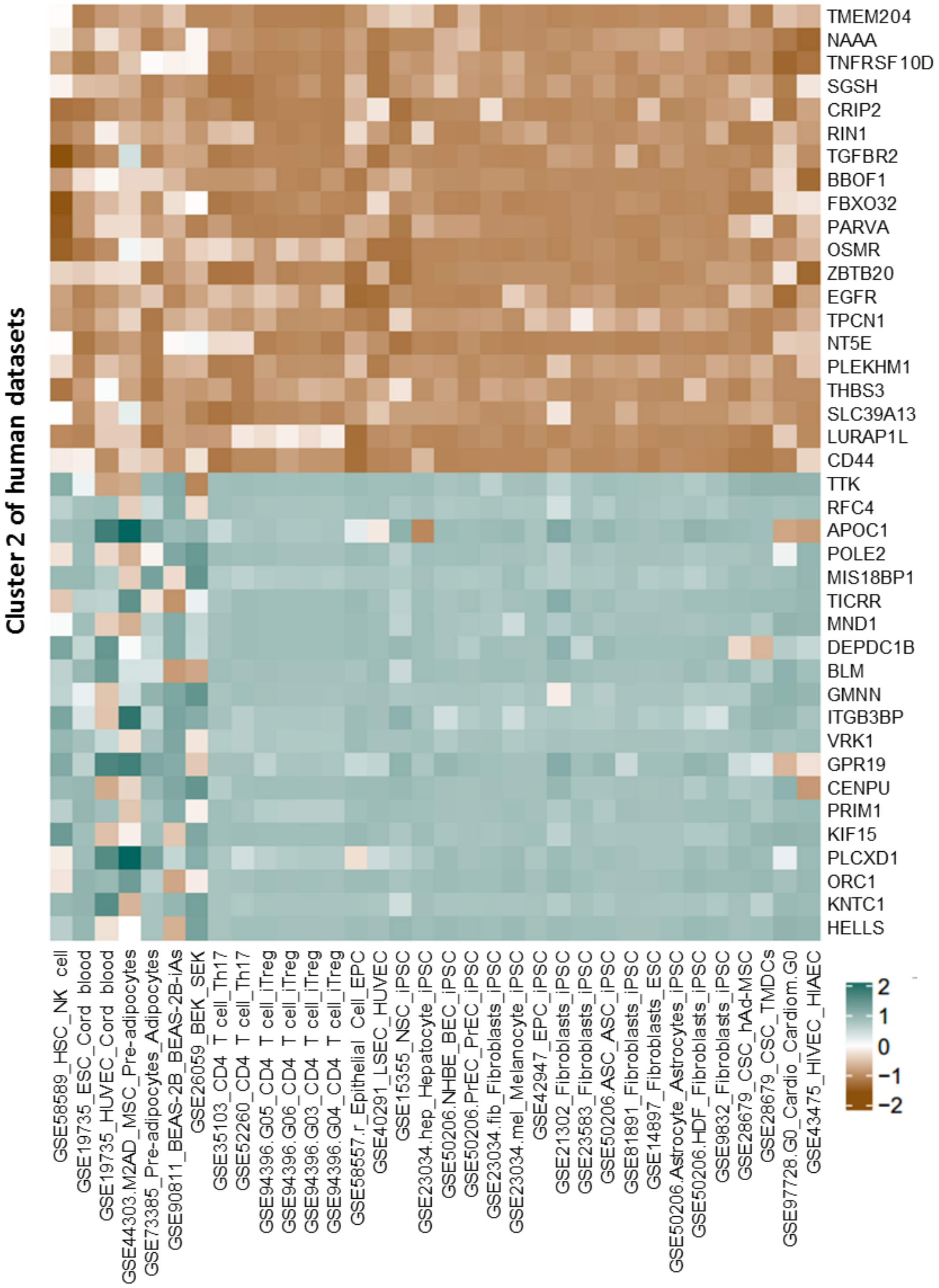

Cluster 2 of human datasets
TMEM204
NAAA
TNFRSF10D
SGSH
CRIP2
RIN1
TGFBR2
BBOF1
FBXO32
PARVA
OSMR
ZBTB20
EGFR
TPCN1
NT5E
PLEKHM1
THBS3
SLC39A13
LURAP1L
CD44
TTK
RFC4
APOC1
POLE2
MIS18BP1
TICRR
MND1
DEPDC1B
BLM
GMNN
ITGB3BP
VRK1
GPR19
CENPU
PRIM1
KIF15
PLCXD1
ORC1
KNTC1
HELLS
GSE58589_HSC_NK_cell
GSE19735_ESC_Cord_blood
GSE19735_HUVEC_Cord_blood
GSE44303.M2AD_MSC_Pre-adipocytes
GSE73385_Pre-adipocytes_Adipocytes
GSE90811_BEAS-2B_BEAS-2B-iAs
GSE26059_BEK_SEK
GSE35103_CD4_T cell_Th17
GSE52260_CD4_T cell_Th17
GSE94396.G05_CD4_T cell_iTreg
GSE94396.G06_CD4_T cell_iTreg
GSE94396.G03_CD4_T cell_iTreg
GSE94396.G04_CD4_T cell_iTreg
GSE58557.r_Epithelial_Cell_EPC
GSE40291_LSEC_HUVEC
GSE15355_NSC_iPSC
GSE23034.hep_Hepatocyte_iPSC
GSE50206.NHBE_BEC_iPSC
GSE50206.PrEC_PrEC_iPSC
GSE23034.fib_Fibroblasts_iPSC
GSE23034.mel_Melanocyte_iPSC
GSE42947_EPC_iPSC
GSE21302_Fibroblasts_iPSC
GSE23583_Fibroblasts_iPSC
GSE50206.ASC_ASC_iPSC
GSE81891_Fibroblasts_iPSC
GSE14897_Fibroblasts_ESC
GSE50206.Astrocyte_Astrocytes_iPSC
GSE50206.HDF_Fibroblasts_iPSC
GSE9832_Fibroblasts_iPSC
GSE28679_CSC_hAd-MSC
GSE28679_CSC_TMDCs
GSE97728.G0_Cardio_Cardiom.G0
GSE43475_HIVEC_HIAEC
2
1
0
−1
−2

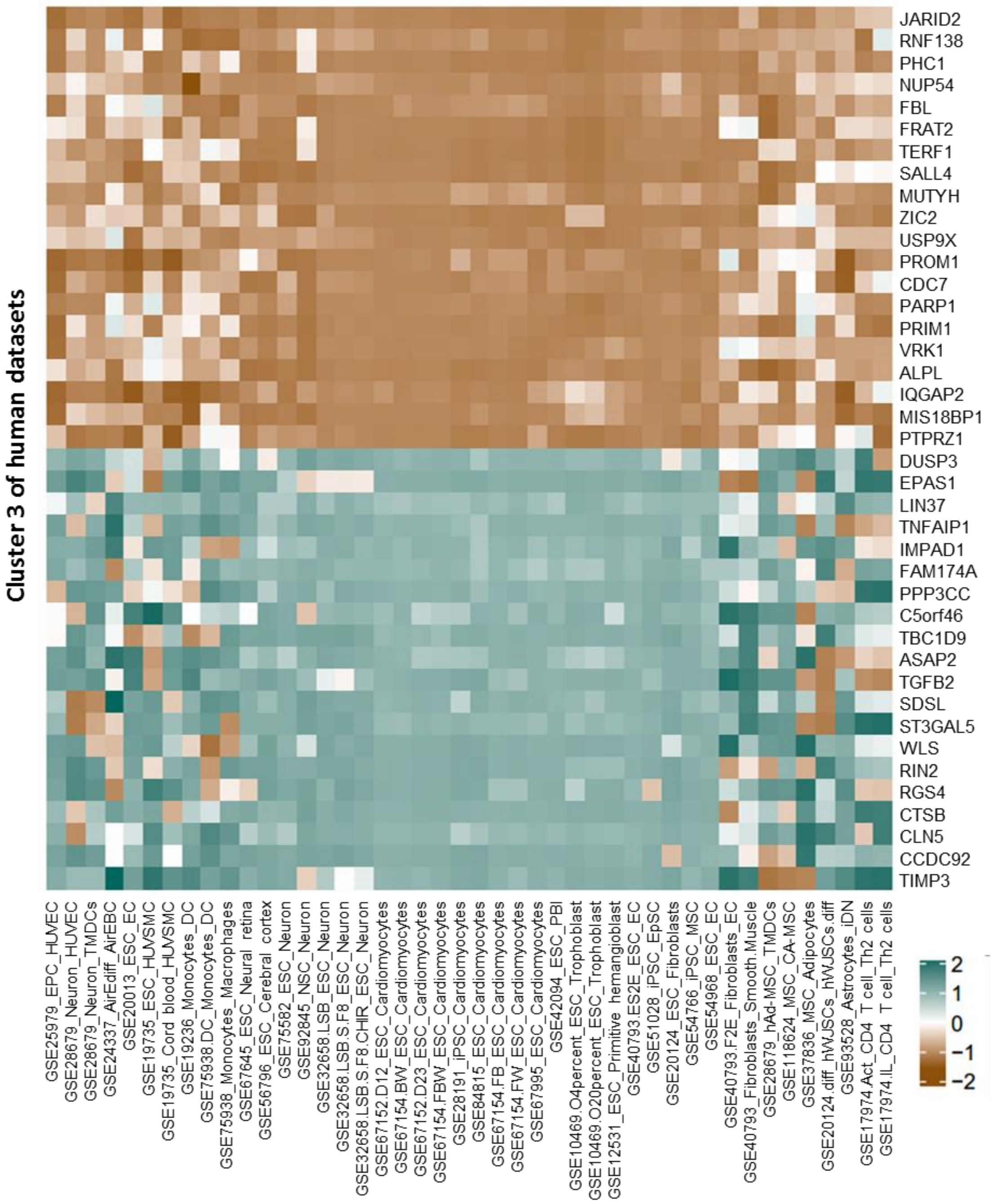
Cluster 3 of human datasets
JARID2
RNF138
PHC1
NUP54
FBL
FRAT2
TERF1
SALL4
MUTYH
ZIC2
USP9X
PROM1
CDC7
PARP1
PRIM1
VRK1
ALPL
IQGAP2
MIS18BP1
PTPRZ1
DUSP3
EPAS1
LIN37
TNFAIP1
IMPAD1
FAM174A
PPP3CC
C5orf46
TBC1D9
ASAP2
TGFB2
SDSL
ST3GAL5
WLS
RIN2
RGS4
CTSB
CLN5
CCDC92
TIMP3
GSE25979_EPC_HUVEC
GSE28679_Neuron_HUVEC
GSE28679_Neuron_TMDCs
GSE24337_AirEdiff_AirEBC
GSE20013_ESC_EC
GSE19735_ESC_HUVSMC
GSE19735_Cord blood_HUVSMC
GSE19236_Monocytes_DC
GSE75938.DC_Monocytes_DC
GSE75938_Monocytes_Macrophages
GSE67645_ESC_Neural retina
GSE56796_ESC_Cerebral cortex
GSE75582_ESC_Neuron
GSE92845_NSC_Neuron
GSE32658.LSB_ESC_Neuron
GSE32658.LSB.S.F8_ESC_Neuron
GSE32658.LSB.S.F8.CHIR_ESC_Neuron
GSE67152.D12_ESC_Cardiomyocytes
GSE67154.BW_ESC_Cardiomyocytes
GSE67152.D23_ESC_Cardiomyocytes
GSE67154.FBW_ESC_Cardiomyocytes
GSE28191_iPSC_Cardiomyocytes
GSE84815_ESC_Cardiomyocytes
GSE67154.FB_ESC_Cardiomyocytes
GSE67154.FW_ESC_Cardiomyocytes
GSE67995_ESC_Cardiomyocytes
GSE42094_ESC_PBI
GSE10469.O4percent_ESC_Trophoblast
GSE10469.O20percent_ESC_Trophoblast
GSE12531_ESC_Primitive hemangioblast
GSE40793.ES2E_ESC_EC
GSE51028_iPSC_EpSC
GSE20124_ESC_Fibroblasts
GSE54766_iPSC_MSC
GSE54968_ESC_EC
GSE40793.F2E_Fibroblasts_EC
GSE40793_Fibroblasts_Smooth.Muscle
GSE28679_hAd-MSC_TMDCs
GSE118624_MSC_CA-MSC
GSE37836_MSC_Adipocytes
GSE20124.diff_hWJSCs_hWJSCs.diff
GSE93528_Astrocytes_iDN
GSE17974.Act_CD4_T cell_Th2 cells
GSE17974.IL_CD4_T cell_Th2 cells
2
1
0
−1
−2

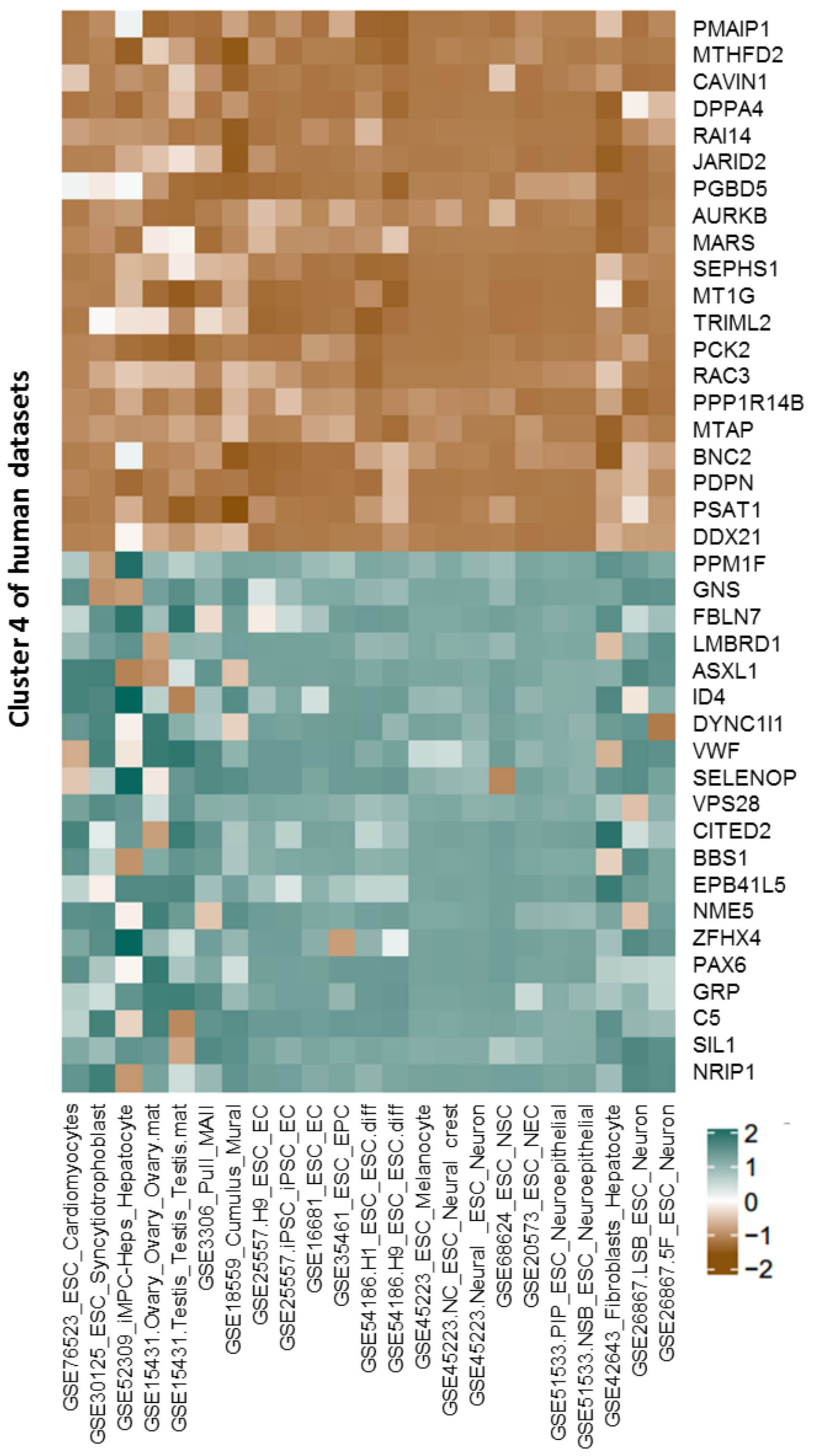
Cluster 4 of human datasets
PMAIP1
MTHFD2
CAVIN1
DPPA4
RAI14
JARID2
PGBD5
AURKB
MARS
SEPHS1
MT1G
TRIML2
PCK2
RAC3
PPP1R14B
MTAP
BNC2
PDPN
PSAT1
DDX21
PPM1F
GNS
FBLN7
LMBRD1
ASXL1
ID4
DYNC1I1
VWF
SELENOP
VPS28
CITED2
BBS1
EPB41L5
NME5
ZFHX4
PAX6
GRP
C5
SIL1
NRIP1
GSE76523_ESC_Cardiomyocytes
GSE30125_ESC_Syncytiotrophoblast
GSE52309_iMPC-Heps_Hepatocyte
GSE15431.Ovary_Ovary_Ovary.mat
GSE15431.Testis_Testis_Testis.mat
GSE3306_Pull_MAII
GSE18559_Cumulus_Mural
GSE25557.H9_ESC_EC
GSE25557.iPSC_iPSC_EC
GSE16681_ESC_EC
GSE35461_ESC_EPC
GSE54186.H1_ESC_ESC.diff
GSE54186.H9_ESC_ESC.diff
GSE45223_ESC_Melanocyte
GSE45223.NC_ESC_Neural crest
GSE45223.Neural _ESC_Neuron
GSE68624_ESC_NSC
GSE20573_ESC_NEC
GSE51533.PIP_ESC_Neuroepithelial
GSE51533.NSB_ESC_Neuroepithelial
GSE42643_Fibroblasts_Hepatocyte
GSE26867.LSB_ESC_Neuron
GSE26867.5F_ESC_Neuron
2
1
0
−1
−2

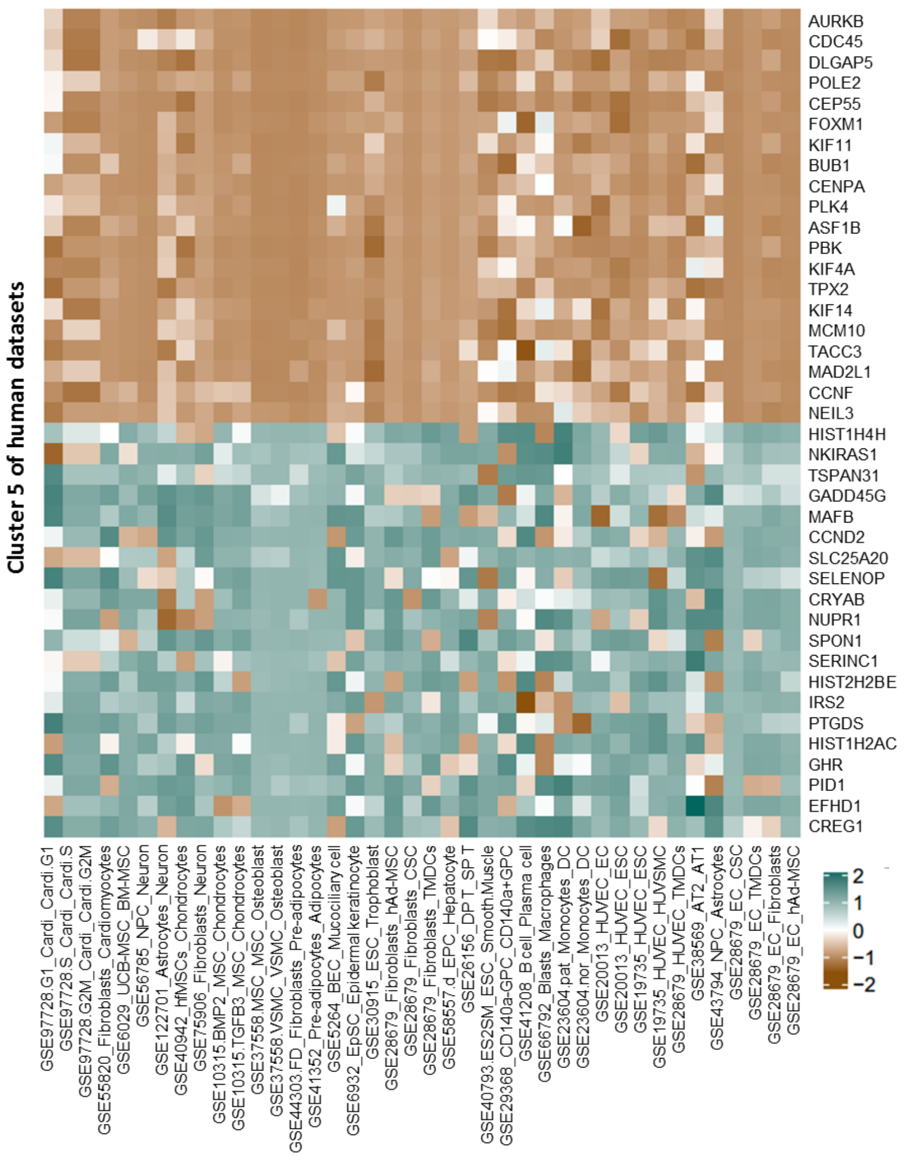

Supplementary Figure 9. Heatmap showing the expression of commonly up-regulated and down-regulated genes in 5 clusters of human datasets.

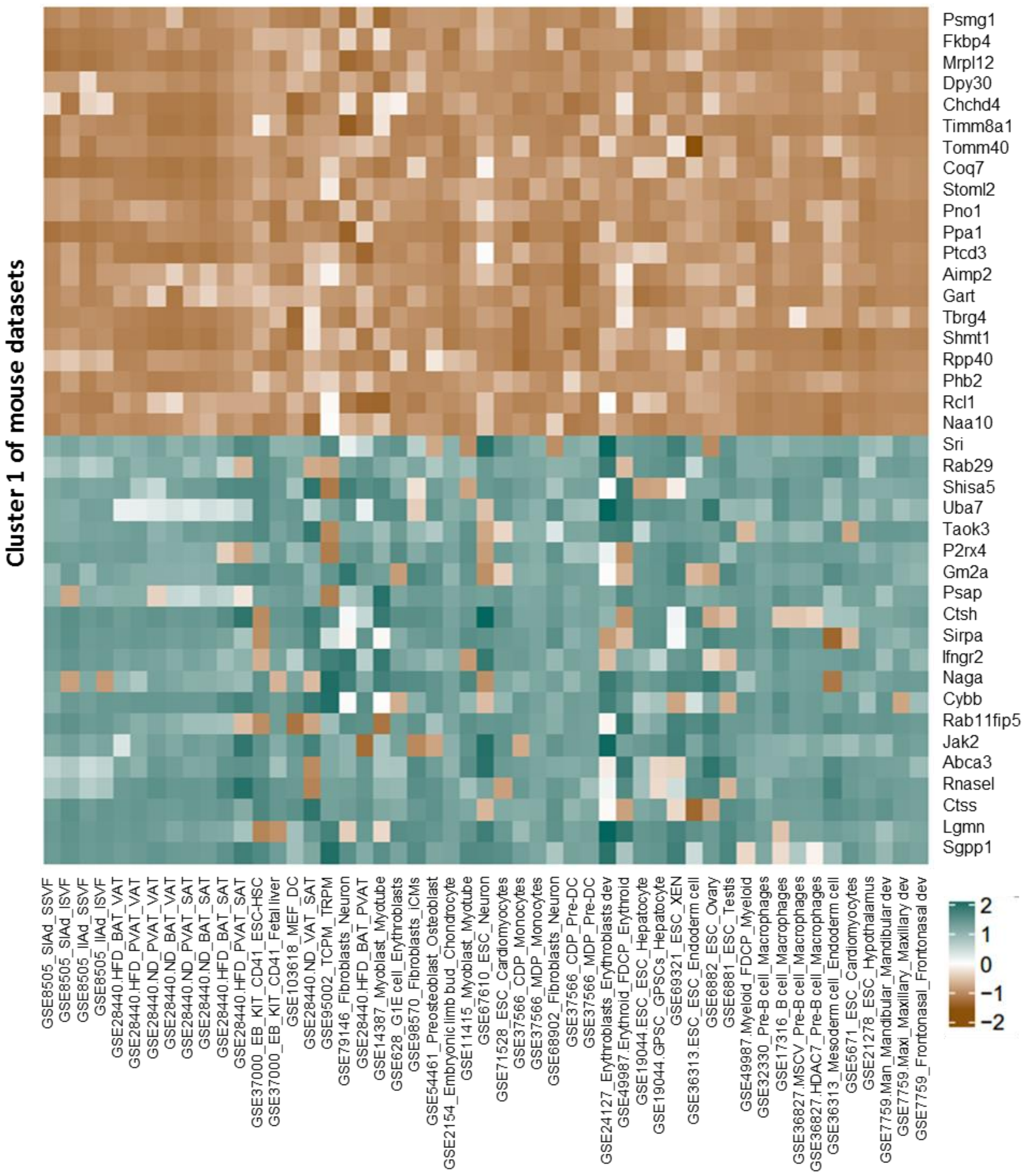
Cluster 1 of mouse datasets
Psmg1
Fkbp4
Mrpl12
Dpy30
Chchd4
Timm8a1
Tomm40
Coq7
Stoml2
Pno1
Ppa1
Ptcd3
Aimp2
Gart
Tbrg4
Shmt1
Rpp40
Phb2
Rcl1
Naa10
Sri
Rab29
Shisa5
Uba7
Taok3
P2rx4
Gm2a
Psap
Ctsh
Sirpa
Ifngr2
Naga
Cybb
Rab11fip5
Jak2
Abca3
Rnasel
Ctss
Lgmn
Sgpp1
GSE8505_SIAd_SSVF
GSE8505_SIAd_ISVF
GSE8505_IIAd_SSVF
GSE8505_IIAd_ISVF
GSE28440.HFD_BAT_VAT
GSE28440.HFD_PVAT_VAT
GSE28440.ND_PVAT_VAT
GSE28440.ND_BAT_VAT
GSE28440.ND_PVAT_SAT
GSE28440.ND_BAT_SAT
GSE28440.HFD_BAT_SAT
GSE28440.HFD_PVAT_SAT
GSE37000_EB_KIT_CD41_ESC-HSC
GSE37000_EB_KIT_CD41_Fetal liver
GSE103618_MEF_DC
GSE28440.ND_VAT_SAT
GSE95002_TCPM_TRPM
GSE79146_Fibroblasts_Neuron
GSE28440.HFD_BAT_PVAT
GSE14387_Myoblast_Myotube
GSE628_G1E cell_Erythroblasts
GSE98570_Fibroblasts_iCMs
GSE54461_Preosteoblast_Osteoblast
GSE2154_Embryonic limb bud_Chondrocyte
GSE11415_Myoblast_Myotube
GSE67610_ESC_Neuron
GSE71528_ESC_Cardiomyocytes
GSE37566_CDP_Monocytes
GSE37566_MDP_Monocytes
GSE68902_Fibroblasts_Neuron
GSE37566_CDP_Pre-DC
GSE37566_MDP_Pre-DC
GSE24127_Erythroblasts_Erythroblasts dev
GSE49987.Erythroid_FDCP_Erythroid
GSE19044.ESC_ESC_Hepatocyte
GSE19044.GPSC_GPSCs_Hepatocyte
GSE69321_ESC_XEN
GSE36313.ESC_ESC_Endoderm cell
GSE6882_ESC_Ovary
GSE6881_ESC_Testis
GSE49987.Myeloid_FDCP_Myeloid
GSE32330_Pre-B cell_Macrophages
GSE17316_B cell_Macrophages
GSE36827.MSCV_Pre-B cell_Macrophages
GSE36827.HDAC7_Pre-B cell_Macrophages
GSE36313_Mesoderm cell_Endoderm cell
GSE5671_ESC_Cardiomyocytes
GSE21278_ESC_Hypothalamus
GSE7759.Man_Mandibular_Mandibular dev
GSE7759.Maxi_Maxillary_Maxillary dev
GSE7759_Frontonasal_Frontonasal dev
2
1
0
−1
−2

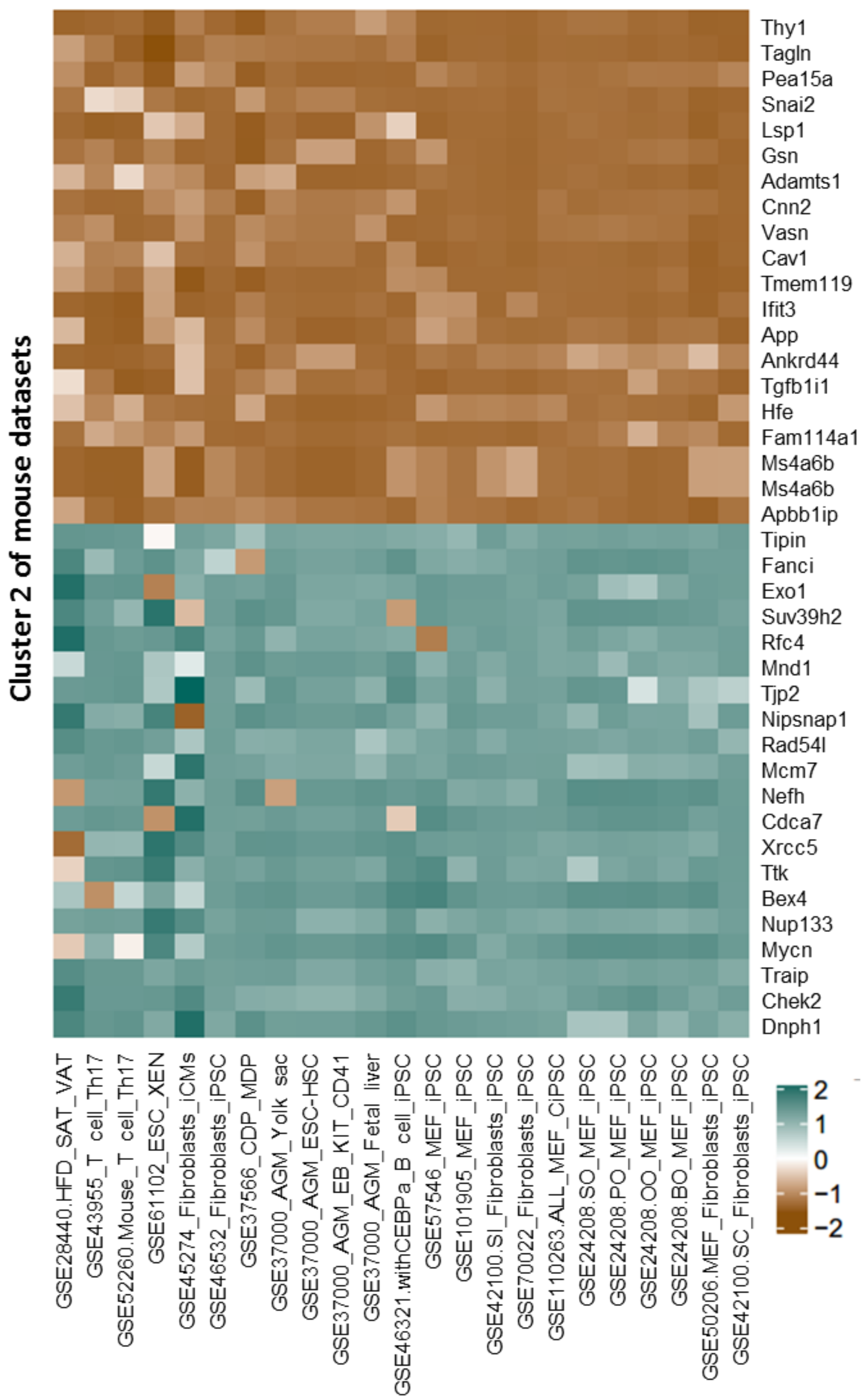

Cluster 2 of mouse datasets
Thy1
Tagln
Pea15a
Snai2
Lsp1
Gsn
Adamts1
Cnn2
Vasn
Cav1
Tmem119
Ifit3
App
Ankrd44
Tgfb1i1
Hfe
Fam114a1
Ms4a6b
Ms4a6b
Apbb1ip
Tipin
Fanci
Exo1
Suv39h2
Rfc4
Mnd1
Tjp2
Nipsnap1
Rad54l
Mcm7
Nefh
Cdca7
Xrcc5
Ttk
Bex4
Nup133
Mycn
Traip
Chek2
Dnph1
GSE28440.HFD_SAT_VAT
GSE43955_T cell_Th17
GSE52260.Mouse_T cell_Th17
GSE61102_ESC_XEN
GSE45274_Fibroblasts_iCMs
GSE46532_Fibroblasts_iPSC
GSE37566_CDP_MDP
GSE37000_AGM_Yolk sac
GSE37000_AGM_ESC-HSC
GSE37000_AGM_EB_KIT_CD41
GSE37000_AGM_Fetal liver
GSE46321.withCEBPa_B cell_iPSC
GSE57546_MEF_iPSC
GSE101905_MEF_iPSC
GSE42100.SI_Fibroblasts_iPSC
GSE70022_Fibroblasts_iPSC
GSE110263.ALL_MEF_CiPSC
GSE24208.SO_MEF_iPSC
GSE24208.PO_MEF_iPSC
GSE24208.OO_MEF_iPSC
GSE24208.BO_MEF_iPSC
GSE50206.MEF_Fibroblasts_iPSC
GSE42100.SC_Fibroblasts_iPSC
2
1
0
−1
−2

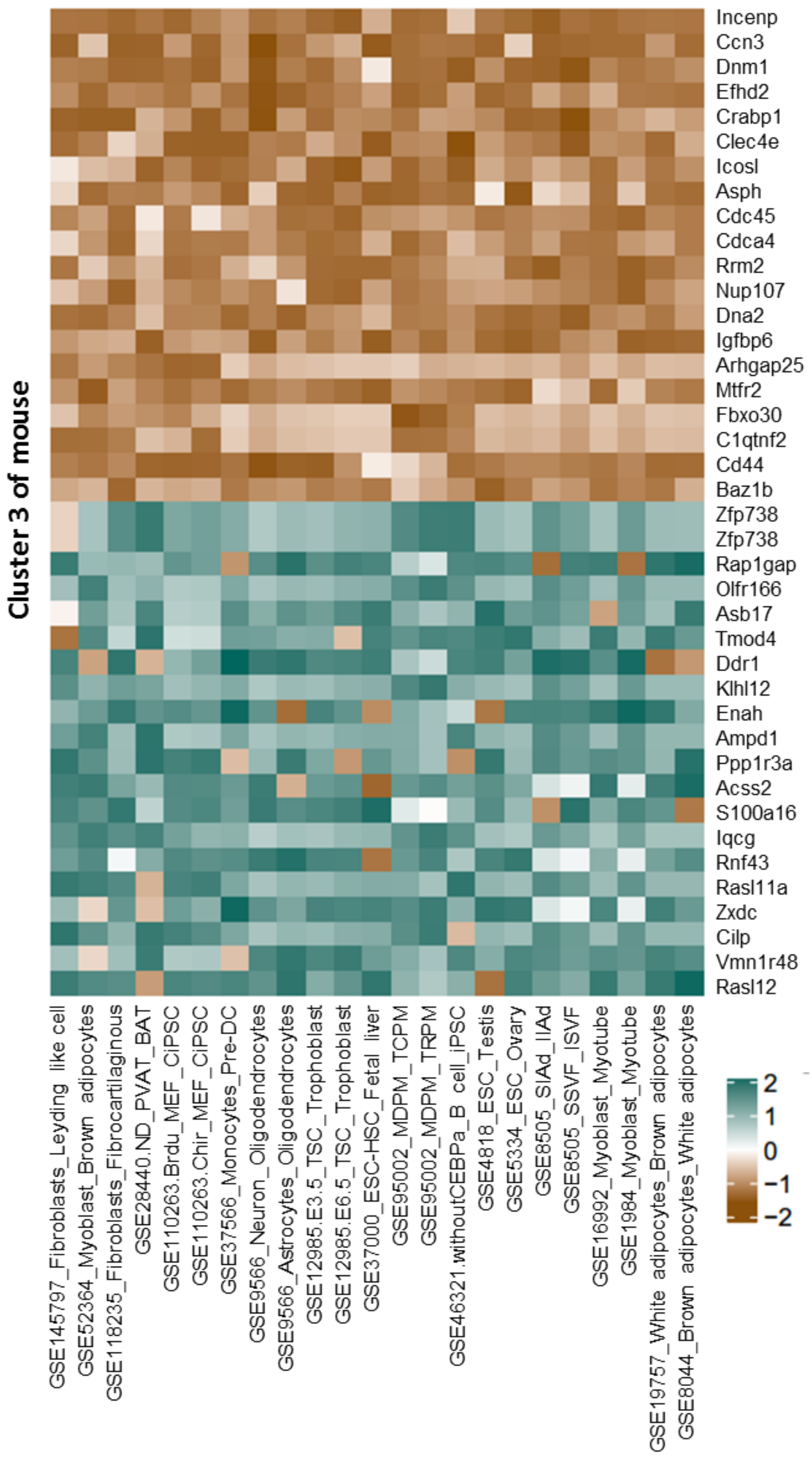
Cluster 3 of mouse
Incenp
Ccn3
Dnm1
Efhd2
Crabp1
Clec4e
Icosl
Asph
Cdc45
Cdca4
Rrm2
Nup107
Dna2
Igfbp6
Arhgap25
Mtfr2
Fbxo30
C1qtnf2
Cd44
Baz1b
Zfp738
Zfp738
Rap1gap
Olfr166
Asb17
Tmod4
Ddr1
Klhl12
Enah
Ampd1
Ppp1r3a
Acss2
S100a16
Iqcg
Rnf43
Rasl11a
Zxdc
Cilp
Vmn1r48
Rasl12
GSE145797_Fibroblasts_Leyding like cell
GSE52364_Myoblast_Brown adipocytes
GSE118235_Fibroblasts_Fibrocartilaginous
GSE28440.ND_PVAT_BAT
GSE110263.Brdu_MEF_CiPSC
GSE110263.Chir_MEF_CiPSC
GSE37566_Monocytes_Pre-DC
GSE9566_Neuron_Oligodendrocytes
GSE9566_Astrocytes_Oligodendrocytes
GSE12985.E3.5_TSC_Trophoblast
GSE12985.E6.5_TSC_Trophoblast
GSE37000_ESC-HSC_Fetal liver
GSE95002_MDPM_TCPM
GSE95002_MDPM_TRPM
GSE46321.withoutCEBPa_B cell_iPSC
GSE4818_ESC_Testis
GSE5334_ESC_Ovary
GSE8505_SIAd_IIAd
GSE8505_SSVF_ISVF
GSE16992_Myoblast_Myotube
GSE1984_Myoblast_Myotube
GSE19757_White adipocytes_Brown adipocytes
GSE8044_Brown adipocytes_White adipocytes
2
1
0
−1
−2

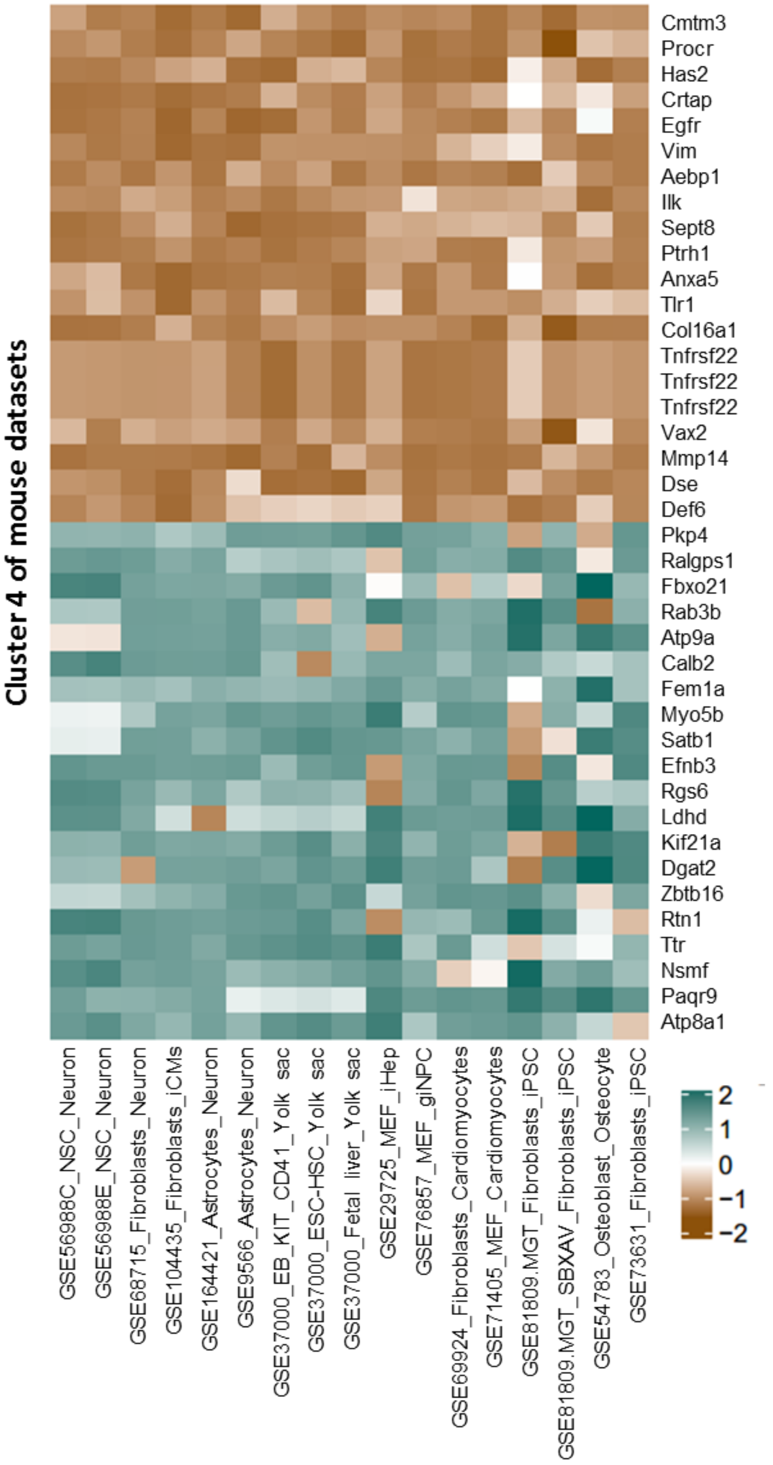

Supplementary Figure 10. Heatmap showing the expression of commonly up-regulated and down-regulated genes in 4 clusters of mouse datasets.

Table 1. Information of the datasets of cell reprogramming from fibroblasts to iPSC.

Datasets	Species	Induction	Time points
GSE81891	Homo sapiens	OSKM	3
GSE89056	Homo sapiens	5iLAF	8
GSE9832	Homo sapiens	OSKM	2
GSE42100	Mus musculus	OSKM	6
GSE50206	Mus musculus	OSKM	8
GSE73631	Mus musculus	VPA, CHIR99021, 616452, tranylcypromine, forskolin and DZNep	6

Table 2. Information of molecules with top similarity scores in the 6 datasets.

Datasets	Molecules	MOA
GSE42100	cycloheximide	Glycogen synthase kinase inhibitor
GSE42100	ibudilast	Leukotriene receptor antagonist
GSE42100	etodolac	Cyclooxygenase inhibitor
GSE42100	GSK-3-inhibitor-IX	Glycogen synthase kinase inhibitor
GSE42100	homoharringtonine	Protein synthesis inhibitor
GSE42100	evodiamine	ATPase inhibitor, TRPV agonist
GSE42100	thioridazine	Dopamine receptor antagonist
GSE42100	valproic-acid	HDAC inhibitor
GSE42100	pinocembrin	CYP1B1 inhibitor
GSE42100	anisomycin	DNA synthesis inhibitor
GSE42100	forskolin	Adenylyl cyclase activator
GSE42100	cycloheximide	Glycogen synthase kinase inhibitor
GSE42100	EPZ004777	Dot1L inhibitor
GSE42100	emetine	Protein synthesis inhibitor
GSE42100	pyroxamide	HDAC inhibitor, Cell cycle inhibitor
GSE42100	genistein	Tyrosine kinase inhibitor
GSE42100	cephaeline	Protein synthesis inhibitor
GSE42100	LY-294002	MTOR inhibitor, PI3K inhibitor
GSE42100	DMEOB	glutamate receptor modulator
GSE42100	cyclopamine	Smoothened receptor antagonist
GSE50206	panobinostat	HDAC inhibitor
GSE50206	trichostatin-a	HDAC inhibitor
GSE50206	cephaeline	Protein synthesis inhibitor
GSE50206	fluoxetine	Serotonin reuptake inhibitor
GSE50206	ciproxifan	NULL
GSE50206	forskolin	Adenylyl cyclase activator
GSE50206	EPZ004777	Dot1L inhibitor

GSE50206	spiperone	Dopamine receptor antagonist
GSE50206	etodolac	Cyclooxygenase inhibitor
GSE50206	dacinostat	HDAC inhibitor
GSE50206	flunisolide	Cytochrome P450 inhibitor
GSE50206	pipamperone	Dopamine receptor antagonist
GSE50206	saracatinib	SRC inhibitor, ABL inhibitor
GSE50206	trichostatin-a	HDAC inhibitor
GSE50206	CAY-10415	Insulin sensitizer
GSE50206	homoharringtonine	Protein synthesis inhibitor
GSE73631	panobinostat	HDAC inhibitor
GSE73631	trichostatin-a	HDAC inhibitor, CDK activator, ID1 inhibitor
GSE73631	cephaeline	Protein synthesis inhibitor
GSE73631	fluoxetine	Serotonin reuptake inhibitor
GSE73631	ciproxifan	NULL
GSE73631	pyrimethamine	Dihydrofolate reductase inhibitor
GSE73631	spiperone	Dopamine receptor antagonist
GSE73631	DNQX	Glutamate receptor antagonist
GSE73631	etodolac	Cyclooxygenase inhibitor
GSE73631	dacinostat	HDAC inhibitor
GSE73631	CHIR-99021	Glycogen synthase kinase inhibitor
GSE73631	lansoprazole	ATPase inhibitor
GSE73631	pipamperone	Dopamine receptor antagonist
GSE73631	EPZ004777	Dot1L inhibitor
GSE73631	risperidone	Dopamine receptor antagonist
GSE73631	trichostatin-a	HDAC inhibitor, CDK activator, ID1 inhibitor
GSE73631	CAY-10415	Insulin sensitizer
GSE73631	homoharringtonine	Protein synthesis inhibitor
GSE81891	quinidine	Cytochrome P450 inhibitor,
GSE81891	pipamperone	Dopamine receptor antagonist
GSE81891	verapamil	Calcium channel blocker
GSE81891	rosiglitazone	PPAR receptor agonist
GSE81891	hydroxyretinoic-acid	Retinoid receptor binder
GSE81891	troglitazone	PPAR receptor agonist
GSE81891	panobinostat	HDAC inhibitor
GSE81891	fulvestrant	Estrogen receptor antagonist
GSE81891	forskolin	Adenylyl cyclase activator
GSE81891	SB-590885	RAF inhibitor
GSE81891	genistein	Tyrosine kinase inhibitor
GSE81891	GSK-3-inhibitor-IX	Glycogen synthase kinase inhibitor
GSE81891	EPZ004777	Dot1L inhibitor
GSE81891	sphingosine	Ceramidase inhibitor
GSE81891	valproic-acid	HDAC inhibitor
GSE81891	medrysone	Glucocorticoid receptor agonist
GSE89056	LE-135	Retinoid receptor agonist

GSE89056	panobinostat	HDAC inhibitor
GSE89056	genistein	Tyrosine kinase inhibitor
GSE89056	PD-184352	MEK inhibitor
GSE89056	lupanine	Sodium channel blocker
GSE89056	acyclovir	DNA polymerase inhibitor
GSE89056	yohimbine	Adrenergic receptor antagonist
GSE89056	BRL-37344	Adrenergic receptor agonist
GSE89056	scriptaid	HDAC inhibitor
GSE89056	CGP-20712	Adrenergic receptor antagonist
GSE89056	EPZ004777	Dot1L inhibitor
GSE89056	erastin	Ion channel antagonist
GSE89056	forskolin	Adenylyl cyclase activator
GSE89056	cyanopindolol	Adrenergic receptor antagonist
GSE89056	SB-590885	RAF inhibitor
GSE89056	bithionol	Autotaxin inhibitor
GSE89056	midodrine	Adrenergic receptor agonist
GSE9832	4-hydroxyretinoic-acid	Retinoid receptor binder
GSE9832	valproic-acid	HDAC inhibitor
GSE9832	glipizide	Sulfonylurea
GSE9832	bemesetron	Serotonin receptor antagonist
GSE9832	CHIR-99021	Glycogen synthase kinase inhibitor
GSE9832	carbidopa	Aromatic L-amino acid decarboxylase inhibitor
GSE9832	EPZ004777	Dot1L inhibitor
GSE9832	fluoxetine	Serotonin reuptake inhibitor
GSE9832	tyramine	Catecholamine releasing agent
GSE9832	pipamperone	Dopamine receptor antagonist
GSE9832	AS-601245	JNK inhibitor
GSE9832	lumicolchicine	Colchicine isomer, non-binder of microtubules

Table 3. Information of 7 molecular compounds.

Compounds	CAS ID	Product ID
Troglitazone	97322-87-7	T3170
Forskolin	66575-29-9	T2939
Valproic-acid	99-66-1	T7064
Carbidopa	28860-95-9	T6795
LY-364947	396129-53-6	T2048
CHIR-99021	252917-06-9	T2310
Panobinostat	404950-80-7	T2383

Supplementary Table 4. The expression of fibroblast-specific genes after molecules treatment (10 uM).

Group	Gene	rep1	rep2	p value	Cohen's D
Control	Acta2	1	1		
Control	Wisp2	1	1		
Troglitazone	Acta2	0.55	0.44	0.012	9.19
Troglitazone	Wisp2	2.75	2.35	0.016	7.75
Forskolin	Acta2	0.15	0.08	0.0016	25.28
Forskolin	Wisp2	0.43	0.55	0.014	8.50
Valproic-acid	Acta2	0.46	0.56	0.01	9.80
Valproic-acid	Wisp2	0.81	0.65	0.078	3.38
Carbidopa	Acta2	0.41	0.51	0.0085	10.80
Carbidopa	Wisp2	0.71	0.61	0.021	6.80
LY-364947	Acta2	0.23	0.15	0.002	20.25
LY-364947	Wisp2	0.11	0.07	0.0005	45.5
CHIR99021	Acta2	0.002	0.08	0.0016	24.59
CHIR99021	Wisp2	0.22	0.11	0.0043	15.18
Panobinostat	Acta2	0.46	0.18	0.05	4.86
Panobinostat	Wisp2	0.55	0.31	0.05	4.75
MPH	Acta2	0.09	0.32	0.02	6.91
MPH	Wisp2	0.13	0.06	0.0015	25.86

Supplementary Table 5. The expression of hepatocyte-specific genes after molecules treatment (10 uM).

Group	Gene	rep1	rep2	p value	Cohen's D
Control	Alb	1	1		
Control	Hnf4a	1	1		
Control	Cyp1a1	1	1		
Control	Cyp2a1	1	1		
Troglitazone	Alb	9.579829	10.33882	0.0018	23.61
Troglitazone	Hnf4a	5.133704	3.89062	0.031	5.65
Troglitazone	Cyp1a1	1.741101	3.09513	0.18	2.09
Troglitazone	Cyp2a1	4.438278	4.85678	0.036	17.43
Forskolin	Alb	304.437	134.3637	0.008	2.57
Forskolin	Hnf4a	10.92832	3.944931	0.21	1.84
Forskolin	Cyp1a1	4.40762	2.42839	0.14	2.44
Forskolin	Cyp2a1	8.876555	3.944931	0.16	2.19
Valproic-acid	Alb	20.9663	33.35891	0.05	4.22
Valproic-acid	Hnf4a	12.04197	11.3924	0.0009	32.99
Valproic-acid	Cyp1a1	4.316913	8.397734	0.12	2.63
Valproic-acid	Cyp2a1	9.000468	8.456144	0.0013	28.4

Carbidopa	Alb	14.12325	22.16175	0.051	4.27
Carbidopa	Hnf4a	9.253506	11.95879	0.02	7.10
Carbidopa	Cyp1a1	3.630077	16.22335	0.29	1.42
Carbidopa	Cyp2a1	7.674113	11.95879	0.055	4.12
LY-364947	Alb	15.24221	18.25222	0.009	10.46
LY-364947	Hnf4a	8.28212	6.19026	0.028	5.96
LY-364947	Cyp1a1	2.42839	5.028053	0.178	2.1
LY-364947	Cyp2a1	7.72749	7.621104	0.001	125.47
CHIR99021	Alb	221.3215	210.8393	0.0006	41.04
CHIR99021	Hnf4a	43.11147	39.12449	0.0025	20.12
CHIR99021	Cyp1a1	24.42015	22.00867	0.003	18.42
CHIR99021	Cyp2a1	34.05984	49.52208	0.034	5.28
Panobinostat	Alb	45.41195	12.005	0.24	1.66
Panobinostat	Hnf4a	8.969329	12.955	0.038	4.99
Panobinostat	Cyp1a1	3.54307	15.455	0.29	1.43
Panobinostat	Cyp2a1	7.917253	17.205	0.13	2.49
MPH	Alb	52498.91	50710.61	0.0003	57.71
MPH	Hnf4a	319.5726	261.3791	0.01	9.95
MPH	Cyp1a1	47.17662	53.81737	0.0045	14.91
MPH	Cyp2a1	694.5814	652.575	0.001	32.02

Supplementary Table 6. The expression of fibroblast-specific genes after molecules treatment (5 uM).

Group	Gene	rep1	rep2	p value	Cohen's D
Control	Acta2	1	1		
Control	Wisp2	1	1		
Troglitazone	Acta2	0.34	0.59	0.051	4.28
Troglitazone	Wisp2	2.3	2.05	0.01	9.4
Forskolin	Acta2	0.17	0.1	0.002	24.71
Forskolin	Wisp2	0.45	0.53	0.006	12.75
Valproic-acid	Acta2	0.42	0.55	0.016	7.92
Valproic-acid	Wisp2	0.95	0.75	0.27	1.5
Carbidopa	Acta2	0.51	0.61	0.013	8.8
Carbidopa	Wisp2	0.78	0.71	0.018	7.29
LY-364947	Acta2	0.21	0.13	0.002	20.75
LY-364947	Wisp2	0.21	0.09	0.005	14.67
CHIR99021	Acta2	0.002	0.1	0.003	19.37
CHIR99021	Wisp2	0.24	0.13	0.0045	14.82
Panobinostat	Acta2	0.49	0.34	0.016	7.8
Panobinostat	Wisp2	0.75	0.52	0.087	3.17
MPH	Acta2	0.05	0.15	0.003	18

MPH	Wisp2	0.11	0.27	0.009	10.12

Supplementary Table 7. The expression of hepatocyte-specific genes after molecules treatment (5 uM).

**Group**	**Gene**	**rep1**	**rep2**	**p value**	**Cohen's D**
Control	Alb	1	1		
Control	Hnf4a	1	1		
Control	Cyp1a1	1	1		
Control	Cyp2a1	1	1		
Troglitazone	Alb	5.94	5.05	0.0095	10.17
Troglitazone	Hnf4a	1.62	2.17	0.082	3.28
Troglitazone	Cyp1a1	0.97	1.69	0.45	0.92
Troglitazone	Cyp2a1	1.28	2.19	0.25	1.63
Forskolin	Alb	140.07	110.66	0.014	8.46
Forskolin	Hnf4a	6.73	4.56	0.05	4.29
Forskolin	Cyp1a1	3.18	4.44	0.047	4.47
Forskolin	Cyp2a1	8.51	5.62	0.05	4.19
Valproic-acid	Alb	18.77	19.84	0.0009	34.21
Valproic-acid	Hnf4a	5.10	5.39	0.0012	29.20
Valproic-acid	Cyp1a1	2.25	4.41	0.16	2.16
Valproic-acid	Cyp2a1	5.35	6.19	0.0076	11.38
Carbidopa	Alb	17.11	21.26	0.013	8.77
Carbidopa	Hnf4a	6.50	4.50	0.046	4.50
Carbidopa	Cyp1a1	2.79	3.97	0.057	4.03
Carbidopa	Cyp2a1	3.86	4.99	0.026	6.03
LY-364947	Alb	17.03	22.94	0.023	6.42
LY-364947	Hnf4a	9.78	8.58	0.0053	13.62
LY-364947	Cyp1a1	5.098	8.75	0.083	3.24
LY-364947	Cyp2a1	10.29	12.91	0.015	8.12
CHIR99021	Alb	101.83	120.26	0.0069	11.94
CHIR99021	Hnf4a	34.06	40.79	0.0084	10.83
CHIR99021	Cyp1a1	23.59	31.56	0.022	6.67
CHIR99021	Cyp2a1	43.41	40.79	0.001	31.31
Panobinostat	Alb	19.56	13.93	0.03	5.59
Panobinostat	Hnf4a	5.43	13.61	0.17	2.08
Panobinostat	Cyp1a1	5.66	14.34	0.17	2.07
Panobinostat	Cyp2a1	9.55	16.77	0.078	3.37
MPH	Alb	52498.91	50710.61	0.0003	57.71
MPH	Hnf4a	319.57	261.38	0.01	9.95
MPH	Cyp1a1	47.18	53.82	0.0045	14.91
MPH	Cyp2a1	694.58	652.58	0.001	32.02

Supplementary Table 8. Abbreviations and full names of cells in PC3T.

Abbreviation	Full name
HFF	Human fetal fibroblasts
hiHep	hepatocyte-like cells
VSMC	Vascular smooth muscle cell
MSC	Mesenchymal Stem cell
AT2	Alveolar epithelial type 2
AT1	Alveolar epithelial type 1
hCBC	Human Cord Blood CD34+ Cells
HUVSMC	Human Umbilical Vein Smooth Muscle Cell
EPC	Endothelial Progenitor Cell
HUVEC	Human umbilical vein endothelial cells
hfMSC	Human fetal bone marrow-derived mesenchymal stromal cell
ESC	Embryonic stem cell
HSC	Hematopoietic stem cell
BEC	Bronchial epithelial cell
NPC	Neural progenitor cells
ASC	Adipose derived stem cell
PrEC	Prostate epithelial cell
TMDCs	Trabecular meshwork-derived cells
EpSC	Epidermal stem cell
NSC	Neural stem cell
DC	Dendritic cell
PHH	Primary human hepatocyte
HPLC	human hepatic progenitor-like cells
CA-MSC	Carcinoma-associated mesenchymal stem cells
iDN	induced dopaminergic neurons
BEAS-2B-iAs	inorganic arsenic-transformed (iAs-T) BEAS-2B
PuII	Pulmonary type II cells
MAII	mature alveolar type II cells
CD140a+GPC	CD140a+ Glial progenitor cells
CD140a-GPC	CD140a- Glial progenitor cells
HHAEC	Human hepatic artery EC
HIAEC	huamn iliac artery EC
HIVEC	human iliac vein EC
HPAEC	human pulmonary artery EC
HUAEC	human umbilical artery EC
HHVEC	human hepatic vein EC
NEC	Neural ectoderm cells
EC	Endothelial cell

PBI	Pancreatic beta Islet
iDN	induced dopaminergic neurons
AirEBC	airway epithelium basal cell
AirEdiff	differentiated airway epithelium
hiPSC	human induced pluripotent stem cells
hiPSC-EpSC	human iPSC-derived epithelial stem cell
BEK	Basal epidermal keratinocytes
SEK	Suprabasal epidermal keratinocytes
hESC-EC	endothelial cells differentiated from H9 human embryonic stem cells
DP T	Double Positive T cell
SP T	Single Positive CD4 T cell
UCB-MSC	umbilical cord blood-derived mesenchymal stem/stromal cell
BM-MSC	bone marrow-derived mesenchymal stem cell
rAC-VEC	reprogrammed amniotic cells induced vascular endothelial cells
CEC	Corneal endothelial cell
LSEC	Liver sinusoidal endothelial cell
iMPC-Heps	induced multipotent progenitor cell from hepatocytes
CSC	Corneal Stromal Cell
HEF	Human embryonic fibroblasts

Supplementary Table 9. Primers used for qRT-PCR.

Gene	Forward Primer (5’ to 3’)	Reverse Primer (5’ to 3’)
Acta2	ACTACTGCCGAGCGTGAGAT	CGTCAGGCAGTTCGTAGCTC
Wisp2	CACGCCCCAGGAGAATACAG	GCAGAAAGTTGGTGTCCTTGG
Alb	TGTGTTGCCGATGAGTCTGC	CGGAGGTTTGGAATGGCACA
Hnf4a	AGACTCCACAGCCATCACCA	AATGGCAGAGGGAGGCTTGA
Cyp1a1	GCCGATCGGAGGTCTTTCTC	AAGACCGCATCTGCACTTGG
Cyp2a1	GACAATGGCGGTCTCATCCC	TGCACGTTAGGCCATGTCAC
β-actin	CCACACTGTGCCCATCTACG	GTGGTGGTGAAGCTGTAGCC
